# Predicting cardiovascular risk from national administrative databases using a combined survival analysis and deep learning approach

**DOI:** 10.1093/ije/dyab258

**Published:** 2021-12-15

**Authors:** Sebastiano Barbieri, Suneela Mehta, Billy Wu, Chrianna Bharat, Katrina Poppe, Louisa Jorm, Rod Jackson

**Affiliations:** Centre for Big Data Research in Health, University of New South Wales, Sydney, NSW, Australia; Section of Epidemiology and Biostatistics, University of Auckland, Auckland, New Zealand; Section of Epidemiology and Biostatistics, University of Auckland, Auckland, New Zealand; National Drug and Alcohol Research Centre, University of New South Wales, Sydney, NSW, Australia; Section of Epidemiology and Biostatistics, University of Auckland, Auckland, New Zealand; Centre for Big Data Research in Health, University of New South Wales, Sydney, NSW, Australia; Section of Epidemiology and Biostatistics, University of Auckland, Auckland, New Zealand

**Keywords:** Cardiovascular diseases, primary prevention, risk assessment, population health, health planning, machine learning, deep learning, survival analysis

## Abstract

**Background:**

Machine learning-based risk prediction models may outperform traditional statistical models in large datasets with many variables, by identifying both novel predictors and the complex interactions between them. This study compared deep learning extensions of survival analysis models with Cox proportional hazards models for predicting cardiovascular disease (CVD) risk in national health administrative datasets.

**Methods:**

Using individual person linkage of administrative datasets, we constructed a cohort of all New Zealanders aged 30–74 who interacted with public health services during 2012. After excluding people with prior CVD, we developed sex-specific deep learning and Cox proportional hazards models to estimate the risk of CVD events within 5 years. Models were compared based on the proportion of explained variance, model calibration and discrimination, and hazard ratios for predictor variables.

**Results:**

First CVD events occurred in 61 927 of 2 164 872 people. Within the reference group, the largest hazard ratios estimated by the deep learning models were for tobacco use in women (2.04, 95% CI: 1.99, 2.10) and chronic obstructive pulmonary disease with acute lower respiratory infection in men (1.56, 95% CI: 1.50, 1.62). Other identified predictors (e.g. hypertension, chest pain, diabetes) aligned with current knowledge about CVD risk factors. Deep learning outperformed Cox proportional hazards models on the basis of proportion of explained variance (R^2^: 0.468 vs 0.425 in women and 0.383 vs 0.348 in men), calibration and discrimination (all *P *<0.0001).

**Conclusions:**

Deep learning extensions of survival analysis models can be applied to large health administrative datasets to derive interpretable CVD risk prediction equations that are more accurate than traditional Cox proportional hazards models.

Key Messages

- This study proposes a combined survival analysis and deep learning approach for cardiovascular disease (CVD) risk prediction, which accounts for censoring of unobserved events and allows estimation of hazard ratios associated with each predictor.
- This study is the first to apply machine learning models for CVD risk prediction across a national population, using predictors available in routinely collected administrative health data.
- The developed models could be used for accurate CVD risk prediction and population health planning in other countries where large administrative health datasets can be linked at the individual person level; for example, they may be used to estimate future CVD incidence, identify target sub-populations for prevention and assess the likely benefit of health policies and interventions in different risk groups.
- The proposed approach has applications beyond CVD risk prediction and could be used in time-to-event analyses to identify diagnoses, procedures and medications associated with other conditions.

## Introduction

Cardiovascular disease (CVD) risk equations, derived in clinical cohorts, are an established means to inform clinical decisions regarding a person’s CVD risk management.^1^ They facilitate risk communication in a clinical setting and motivate adherence to recommended treatment and lifestyle modifications.^2^ A complementary use of CVD risk equations is their derivation in routine administrative datasets and their application to every person in a given population for population health planning (e.g. estimation of future CVD incidence, identification of target sub-populations for prevention and assessment of the likely benefit of health policies and interventions in different risk groups).^3^^,^^4^ We have previously developed equations to estimate the 5-year risk of a fatal or non-fatal CVD event across the primary prevention population of New Zealand, solely using linked routine national administrative health datasets, and these equations showed good calibration and discrimination across risk groups stratified by age, ethnicity, geographical region, level of deprivation and previous CVD-related pharmaceutical dispensing.^5^

CVD risk equations for population health planning differ from equations in clinical use as they can only consider predictors available in routinely collected administrative health data, which usually do not include smoking status, blood pressure and lipid profile. However, administrative health data may contain useful proxies for missing CVD predictors, e.g. diagnoses and procedures recorded during hospitalizations and pharmaceutical dispensing. If additional CVD predictors can be identified in routinely collected data, the predictive accuracy of CVD risk equations for population health planning can be improved. Whereas traditional methods for statistical inference using longitudinal data, such as Cox proportional hazards regression,^6^ require predictors to be pre-specified and become less reliable as the number of predictors and possible associations among them increase,^7^ machine learning can be used to identify relevant patterns across complex multimodal data. Recent methodological developments, which replaced the linear combination of predictors in a Cox proportional hazards model with a deep neural network, were able to demonstrate improved calibration and discrimination results.^8^ In this study, deep learning extensions of survival analysis models were applied to routinely collected administrative health data to predict the 5-year CVD risk of over two million adult New Zealanders.

This study had the following aims: (i) to develop novel deep learning models for predicting the 5-year risk of a fatal or non-fatal CVD event across the New Zealand adult population without prior CVD or heart failure, using routinely collected administrative health data; (ii) to determine which diagnoses, procedures and dispensed medications are associated with increased risk of CVD event; (iii) to compare the performance of the deep learning models and traditional Cox proportional hazards models on the basis of the proportion of explained variance, calibration and discrimination.

## Methods

### Study population and data sources

This study has been performed in accordance with the ethical standards laid down in the 1964 Declaration of Helsinki and is part of the VIEW research programme, which was approved by the Northern Region Ethics Committee Y in 2003 (AKY/03/12/314), with subsequent annual re-approval by the national Multi-Region Ethics Committee since 2007 (MEC/01/19/EXP). Individual patient consent was not required, as all data are anonymized.

The study population comprised New Zealand residents aged 30–74 years who were alive, in New Zealand, on 31 December 2012 (index date) and with a health contact recorded in one or more of the following New Zealand routine national health databases: demographic characteristics, primary care enrolment (with voluntary re-enrolment occurring every 3 years), primary care visit reimbursements (to capture primary care visits by non-enrolled patients), community laboratory requests (but no laboratory results), community pharmaceutical dispensing, outpatient visits, hospitalizations and deaths. Data for each person were linked based on the National Health Index number (NHI number), a unique identifier assigned to every person who uses health and disability support services in New Zealand (estimated 98% of the population^9^). NHI numbers in the linked dataset were encrypted at source and all other individual patient identifiers were also removed. The dataset was linked to the Virtual Diabetes Registry, administered by the New Zealand Ministry of Health, to identify individuals with a history of diabetes as at 31 December 2012. The age range for inclusion reflects the age group recommended for CVD risk assessment in New Zealand.^10^

People with a history of CVD, heart failure or missing predictor variables were excluded from the dataset. People were considered to have a history of CVD or heart failure if their data contained relevant ICD10-AM codes associated with hospitalizations between 1 January 1993 and the index date, if they were dispensed loop diuretics or antianginals three or more times in the 5 years prior to the index date or if they were dispensed metolazone in the 6 months prior to the index date. The cohort development flowchart is presented in Supplementary Figure S1, available as Supplementary data at *IJE* online. Additional information is reported in Supplementary Methods, available as Supplementary data at *IJE* online.

### Outcome

The outcome of interest was the time in days to the first fatal or non-fatal CVD event identified from national hospitalization and mortality datasets over the 5-year period between 1 January 2013 and 31 December 2017 (ICD10-AM codes are reported in the Supplementary Methods). Five years is the recommended CVD risk prediction period in New Zealand guidelines.^10^ People who died of CVD-unrelated causes during follow-up were censored. People who ceased to have any recorded health contact before 31 December 2017 were assumed to have left New Zealand during follow-up and were also censored at their last recorded contact date with a health provider.

### Deep learning models

#### Predictors

Deep learning models for predicting 5-year CVD risk were developed using linked data for the described 2012 study population.^5^ The input data contained both pre-specified predictors and all diagnoses, procedures and medications in the 5 years prior to the index date.

Pre-specified predictors were included to facilitate comparisons between models. These pre-specified predictors were selected based on evidence regarding CVD risk factors and availability in the national health databases, and included: sex, age, ethnicity, level of deprivation, diabetes status, previous hospitalization for atrial fibrillation (including both primary and secondary diagnoses between 1 January 1993 and the index date) and baseline dispensing of blood-pressure-lowering, lipid-lowering and antiplatelet/anticoagulant medications, respectively. Deprivation was available in national health databases according to deciles of the New Zealand Index of Deprivation 2006 (NZDep2006), but was aggregated into quintiles (i.e. 1–5) to mitigate the effect of reassignment between deciles which occurs with different versions of the deprivation index over time. Age and deprivation quintiles were centred for analysis, using the mean value for age and the third quintile for level of deprivation. Changes in CVD pharmacotherapy over 5 years have been shown to be infrequent^11^ and were not considered. First-order interaction terms were included based on clinical plausibility and statistical significance in traditional Cox proportional hazards models (*P*-value of <0.001).^11^ Additional information regarding the pre-specified predictors is available in the Supplementary Methods.

ICD10-AM coded diagnoses and procedures and dispensed medications were sorted chronologically by year and calendar month. Whenever a person’s hospitalization or medication period spanned multiple calendar months, the associated diagnoses, procedures and medications were listed a corresponding number of times. Each listed ICD10-AM code was also associated with a variable indicating whether it was a primary diagnosis, a secondary diagnosis, an external cause of injury or a procedure/operation. Rare ICD10-AM codes and medications associated with less than 500 people were excluded.

#### Neural network architecture

**Figure 1:**
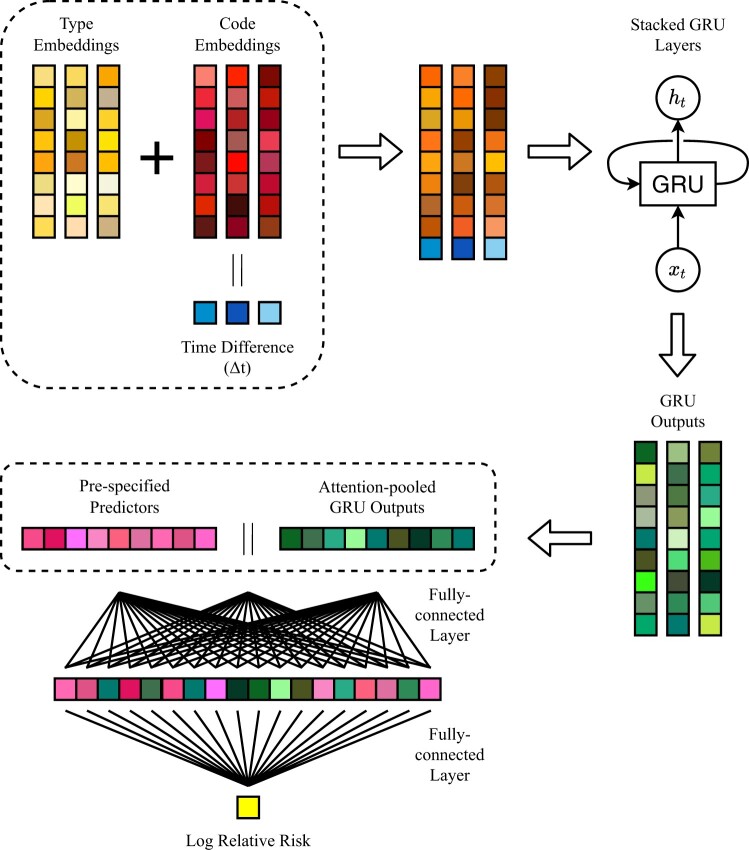
A schematic representation of the neural network used to map a person’s predictors and clinical history to the log of the relative risk function. Code embeddings indicate vector representations of diagnoses, procedures and medications. Type embeddings describe the type of code (primary diagnosis, secondary diagnosis, external cause of injury, or procedure or operation). An extended description is reported in the main text. The ‘‖’ symbol indicates vector concatenation. Gated recurrent units (GRUs) are a gating mechanism in recurrent neural networks

A schematic representation of the neural network used to map a person’s pre-specified predictors, diagnoses, procedures and medications to the log of the relative risk function is presented in Figure 1. At first, ICD10-AM codes and medications are represented as high-dimensional real-valued vectors (embeddings). The embeddings for ICD10-AM codes are summed with other embeddings describing the type of code (primary diagnosis, secondary diagnosis, external cause of injury or procedure/operation). These vectors are then concatenated with a scalar value Δt, indicating the time difference, in months, between the current and the previous code in the clinical history (Δt = 0 for the first code). Next, the vectors are passed to three stacked bidirectional gated recurrent unit (GRU) layers with 10% dropout. GRUs are a gating mechanism in recurrent neural networks; for each sequential input vector, a GRU outputs a vector which depends on the current input and the GRU’s internal state (memory). Therefore, they are able to generate a vector representation of an input code in the context of a person’s recent clinical history. The size of the GRU’s hidden state was set equal to the input size. Three stacked layers were used to increase the network’s expressive power. To focus on the most relevant outputs of the GRU layers and to obtain a single vector representation of the entire clinical history, a linear combination of the outputs was computed using dot-product attention. The resulting vector was concatenated with the pre-specified predictors, passed through a size-preserving fully connected layer with exponential linear unit (ELU) activation and finally mapped to a scalar value (the log of the relative risk) by another fully connected layer. Network hyperparameters were optimized using 10% of the data, stratified by outcome; details and additional references are reported in the Supplementary Methods.

#### Training

The remaining 90% of the data were used to train and test the deep learning models, using stratified 5 × 2 cross-validation.^12^ Sex-specific estimates of network parameters were obtained by maximizing the Cox partial likelihood on the training data, using mini-batch stochastic gradient descent.^8^ An Adam optimizer with a learning rate of 0.001 and β = (0.9, 0.999) was used for stochastic optimization.^13^ Training epochs iterated over all people who experienced a CVD event, and individual mini-batches consisted of 256 cases matched one-to-one with random controls in the respective risk set.^8^ During hyperparameter optimization, overfitting of the training data became apparent after approximately 10 training epochs and therefore training was stopped after 10 epochs. An ensemble of 10 neural networks was constructed for each cross-validation fold by repeating training with different random parameter initializations and averaging predictions.^14^

Five-year risk predictions were derived by estimating the baseline survival at the mean value of age, the third quintile of level of deprivation and the reference group of categorical variables.^15^

#### Testing and validation in New Zealand sub-populations

The deep learning models were evaluated quantitatively based on Royston and Sauerbrei’s R^2^ (a measure of the proportion of explained variance^16^), Royston and Sauerbrei’s D statistic,^16^ Harrell’s C statistic^17^ and the integrated Brier score.^18^ Royston and Sauerbrei’s D statistic and Harrell’s C statistic are measures of discrimination. Royston and Sauerbrei’s D statistic represents the log hazard ratio of two equally sized prognostic groups identified by dividing the study population according to the median of the prognostic index. Therefore, the D statistic quantifies the prognostic separation of survival curves between these two groups.^16^ Harrell’s C statistic estimates the proportion of pairs of individuals where concordance is observed between predictions and outcomes.^17^ The expected Brier score may be interpreted as the mean square error of prediction, and is affected by both calibration and discrimination.^18^^,^^19^ The integrated Brier score averages model performance over all available times. Further qualitative assessment was performed through calibration plots and discrimination plots (i.e. dot charts of proportion of events occurring in each decile of predicted risk). Qualitative assessment was repeated for New Zealand sub-populations stratified by: (i) 15-year age bands; (ii) ethnicity; (iii) quintiles of deprivation; and (iv) dispensing of preventive medications.

#### Statistical inference

To approximate the uncertainty of network parameters, the deep learning models were trained on the entire dataset 100 times with different random parameter initializations.^14^ For each trained model, a baseline risk was computed for a person of mean age, in the third quintile of level of deprivation, in the reference group of categorical variables and with no associated diagnoses, procedures or medications. The data for this person was then perturbed by either changing the values of continuous or binary predictors, or adding an individual diagnosis, procedure or medication. The resulting change in risk was used to estimate sex-specific ‘local hazard ratios’ (HRs) for the modified predictor. Local HRs averaged across trained models were reported together with 95% confidence intervals (CI). The local HRs are valid only for comparisons with the selected baseline population, whereas HRs derived by traditional Cox proportional hazards models describe general changes in hazard when other predictors are kept constant.

### Comparison with Cox proportional hazards models

Sex-specific, multivariable Cox proportional hazards CVD risk models were developed using the same pre-specified predictors and first-order interaction terms used to develop the deep learning models. The methodology for developing the Cox models has been described previously in detail.^5^ For the current study, these models were replicated in the 2012 New Zealand population. As for the deep learning models, calibration and discrimination measures were computed using 5 × 2 cross-validation. The statistical significance of differences between the deep learning and the traditional Cox models was assessed using combined 5 × 2 *F* tests.^12^ Hazard ratios were determined after fitting the Cox models to the entire dataset.

### Neural network ablation study

The predictors used to develop the deep learning models were partly redundant: diabetes status, previous hospitalization for atrial fibrillation and baseline dispensing of blood-pressure-lowering, lipid-lowering and antiplatelet/anticoagulant medications were included both as binary predictors and as individual ICD10-AM codes or medications. Similarly, first-order interaction terms between pre-specified predictors were part of the input but could also have been computed by the fully connected layers of the deep learning models. Therefore, the deep learning models were re-trained using only age, ethnicity and level of deprivation as pre-specified predictors. Local HRs, the proportion of explained variance, and calibration and discrimination measures were computed for these ‘deep learning models without redundant predictors’ and compared with the deep learning models using all pre-specified predictors.

### Hardware and software

The deep learning models were implemented in Python 3.7.5 using PyTorch 1.5.1 and based in part on the PyCox library.^8^ Hyperparameter optimization was performed using Optuna 1.5.0. One training epoch took approximately 90 s on a personal computer with a 3.50 GHz Intel Xeon processor, 64 GB of random access memory and a NVIDIA GeForce RTX 2080 Super graphics card. Statistical analyses to develop the Cox proportional hazards models were undertaken using Stata software version 14.1. The developed algorithms, trained deep learning models and tabulated results are publicly available at [https://github.com/VIEW2020/Varianz2012].

## Results

After applying the exclusion criteria (Supplementary Figure S1), the cohort for this study comprised 2 164 872 New Zealand residents aged 30–74 years and still alive on 31 December 2012 (Table 1). The proportion of women was 52.7%. The majority of the study population was of European (69.8% of women and 71.8% of men) and Māori (11.6% of women and 10.5% of men) descent, with 5.3% Pacific peoples, 3.5% Indian and 9.3% of other or unknown descent. The estimated prevalence of diabetes was around 6% for both sexes, and 0.6% of women and 1.2% of men had recorded diagnoses of atrial fibrillation. Blood-pressure-lowering medications were the most commonly dispensed category of baseline CVD preventive pharmacotherapy (17.0% of women, 16.4% of men).

**Table 1:** Participant characteristics (*N *= 2 164 872)

	Women^a^	Men^a^
Participants	1 141 925 (52.7%)	1 022 947 (47.3%)
Age in years, mean (standard deviation)	49.0 (11.8)	49.0 (11.6)
Ethnicity		
European	797 571 (69.8%)	734 891 (71.8%)
Māori	132 802 (11.6%)	106 912 (10.5%)
Pacific	60 965 (5.3%)	54 659 (5.3%)
Indian	38 481 (3.4%)	36 248 (3.5%)
Other	112 106 (9.8%)	90 237 (8.8%)
Deprivation quintile		
1	272 564 (23.9%)	242 794 (23.7%)
2	244 140 (21.4%)	216 602 (21.2%)
3	227 684 (19.9%)	202 118 (19.8%)
4	212 257 (18.6%)	190 774 (18.6%)
5	185 280 (16.2%)	170 659 (16.7%)
Diabetes	67 143 (5.9%)	65 290 (6.4%)
Atrial fibrillation	6393 (0.6%)	11 900 (1.2%)
Medications dispensed at baseline		
Blood-pressure-lowering	194 670 (17.0%)	167 839 (16.4%)
Lipid-lowering	110 428 (9.7%)	137 529 (13.4%)
Antiplatelet/anticoagulant	64 158 (5.6%)	79 443 (7.8%)
Follow-up		
Total follow-up, years (mean)	5 451 552 (4.8)	4 792 390 (4.7)
Cardiovascular disease deaths	2986 (0.3%)	5153 (0.5%)
Cardiovascular disease events (non-fatal and fatal)	23 592 (2.1%)	38 335 (3.7%)
Median time to cardiovascular disease event, years^b^ (interquartile range)	2.8 (1.4, 3.9)	2.7 (1.4, 3.9)
Non-cardiovascular disease deaths	13 771 (1.2%)	15 660 (1.5%)
Censored at 5 years	1 021 829 (89.5%)	866 167 (84.7%)

a Values are *N* (%) unless otherwise stated.

b Among those with an event between 2013 and 2017 inclusively.

Among the women in this study, 2.1% experienced a CVD event during a mean follow-up time of 4.8 years (0.3% experienced a fatal CVD event). Among men, 3.7% experienced a CVD event during a mean follow-up time of 4.7 years (0.5% experienced a fatal CVD event).

### Deep learning models

Tables 2 and 3 present the adjusted local hazard ratios for predictors in the sex-specific deep learning models, together with the proportions of people with each risk factor. Only the 10 diagnoses and procedures and the 10 medications associated with the largest local hazard ratios are reported; the full tables can be accessed at [https://github.com/VIEW2020/Varianz2012].

**Table 2:** Adjusted local hazard ratios (HRs) for time to cardiovascular disease event within 5 years for women, determined by the deep learning model (only the 10 diagnoses and procedures, and the 10 medications, associated with the largest hazard ratios are reported)

Women (*N *= 1 141 925)		Deep learning model
Predictors	*N* (%)	Adjusted local HRs (95% CI)^a^
Age (per year)^b^		1.09 (1.06, 1.11)^c^
Ethnicity		
European	797 571 (69.8%)	1
Māori	132 802 (11.6%)	1.96 (1.95, 1.97)
Pacific	60 965 (5.3%)	1.68 (1.67, 1.69)
Indian	38 481 (3.4%)	0.925 (0.918, 0.932)
Other	112 106 (9.8%)	0.720 (0.716, 0.723)
Deprivation quintile (per quintile)^b^		1.16 (1.15, 1.16)^c^
Diabetes	67 143 (5.9%)	1.39 (1.37, 1.40)
Atrial fibrillation	6393 (0.6%)	1.68 (1.66, 1.69)
Medications dispensed at baseline		
Blood pressure lowering	194 670 (17.0%)	1.31 (1.29, 1.33)
Lipid lowering	110 428 (9.7%)	0.998 (0.990, 1.01)
Antiplatelet/anticoagulant	64 158 (5.6%)	1.46 (1.45, 1.47)
Interactions		
Age (years)*blood-pressure-lowering medication		0.980 (0.978, 0.982)
Age (years)*diabetes		0.999 (0.997, 1.00)
Age (years)*atrial fibrillation		0.963 (0.961, 0.966)
Blood-pressure-lowering medication*diabetes		1.10 (1.09, 1.11)
Antiplatelet/anticoagulant medications*diabetes		0.883 (0.874, 0.892)
Blood-pressure-lowering medication*lipid -lowering medication		0.997 (0.989, 1.01)
Top 10 diagnoses and procedures		
Z72.0: Tobacco use, current	84 589 (7.4%)	2.04 (1.99, 2.10)
I10: Essential (primary) hypertension	14 167 (1.2%)	1.98 (1.91, 2.06)
R07.4: Chest pain, unspecified	17 208 (1.5%)	1.69 (1.63, 1.76)
92514-39: General anaesthesia, ASA 3 (Patient with severe systemic disease that limits activity), nonemergency or not known	10 961 (1.0%)	1.55 (1.49, 1.61)
56001-00: Computerized tomography of brain	16 845 (1.5%)	1.53 (1.47, 1.58)
J44.1: Chronic obstructive pulmonary disease with acute exacerbation, unspecified	1096 (0.1%)	1.52 (1.47, 1.58)
Z92.2: Personal history of long-term (current) use of other medicaments	2661 (0.2%)	1.52 (1.47, 1.58)
H35.0: Background retinopathy and retinal vascular changes	692 (0.1%)	1.51 (1.46, 1.57)
Z92.22: Personal history of long-term (current) use of other medicaments, insulin	2169 (0.2%)	1.47 (1.42, 1.53)
Z72.1: Alcohol use	957 (0.1%)	1.45 (1.40, 1.50)
Top 10 medications		
Nicotine	79 506 (7.0%)	1.74 (1.70, 1.78)
Varenicline tartrate	31 750 (2.8%)	1.54 (1.50, 1.58)
Furosemide]	13 340 (1.2%)	1.44 (1.40, 1.49)
Tiotropium bromide	4078 (0.4%)	1.43 (1.39, 1.47)
Bupropion hydrochloride	30 796 (2.7%)	1.40 (1.36, 1.43)
Cilazapril	76 762 (6.7%)	1.38 (1.35, 1.41)
Malathion	22 441 (2.0%)	1.37 (1.33, 1.41)
Salbutamol with ipratropium bromide	22 240 (1.9%)	1.35 (1.32, 1.39)
Quinapril	48 373 (4.2%)	1.33 (1.30, 1.37)
Glyceryl trinitrate	15 899 (1.4%)	1.31 (1.26, 1.37)

a The local hazard ratios for each predictor are adjusted for all other predictors. Values in parentheses are 95% confidence intervals unless otherwise stated.

b Age was centred at the mean value of 49.021. Deprivation quintile was centred around quintile three. The baseline survival estimate at 5 years for the deep learning model, relevant to the mean value of age, deprivation quintile three and the reference group of categorical variables, was 0.9926104519395.

c Average and range (in parentheses) of estimated local hazard ratios for all values of the continuous predictor.

**Table 3:** Adjusted local hazard ratios (HRs) for time to cardiovascular disease event within 5 years for men, determined by the deep learning model (only the 10 diagnoses and procedures and the 10 medications associated with the largest hazard ratios are reported)

Men (*N* = 1 022 947)		Deep learning model
Predictors	*N* (%)	Adjusted local HRs (95% CI)^a^
Age (per year)^b^		1.09 (1.06, 1.13)^c^
Ethnicity		
European	734 891 (71.8%)	1
Māori	106 912 (10.5%)	1.69 (1.69, 1.70)
Pacific	54 659 (5.3%)	1.44 (1.43, 1.44)
Indian	36 248 (3.5%)	1.40 (1.39, 1.41)
Other	90 237 (8.8%)	0.785 (0.781, 0.790)
Deprivation quintile (per quintile)^b^		1.10 (1.09, 1.10)^c^
Diabetes	65 290 (6.4%)	1.46 (1.45, 1.47)
Atrial fibrillation	11 900 (1.2%)	1.61 (1.59, 1.62)
Medications dispensed at baseline		
Blood-pressure-lowering	167 839 (16.4%)	1.12 (1.11, 1.13)
Lipid-lowering	137 529 (13.4%)	0.937 (0.929, 0.945)
Antiplatelet/anticoagulant	79 443 (7.8%)	1.43 (1.42, 1.44)
Interactions		
Age (years)*blood-pressure-lowering medication		0.987 (0.986, 0.989)
Age (years)*diabetes		0.993 (0.991, 0.995)
Age (years)*atrial fibrillation		0.994 (0.991, 0.996)
Blood-pressure-lowering medication*diabetes		0.969 (0.960, 0.978)
Antiplatelet/anticoagulant medications*diabetes		0.855 (0.848, 0.863)
Blood-pressure-lowering medication*lipid-lowering medication		1.01 (1.01, 1.02)
Top 10 diagnoses and procedures		
J44.0: Chronic obstructive pulmonary disease with acute lower respiratory infection	1529 (0.1%)	1.56 (1.50, 1.62)
N18.90: Unspecified chronic renal failure	909 (0.1%)	1.54 (1.49, 1.60)
R07.3: Other chest pain	7665 (0.7%)	1.51 (1.45, 1.57)
E11.71: Non-insulin-dependent diabetes mellitus with multiple complications, stated as uncontrolled	663 (0.1%)	1.51 (1.45, 1.56)
L97: Ulcer of lower limb, not elsewhere classified	896 (0.1%)	1.50 (1.46, 1.55)
E11.72: Type 2 diabetes mellitus with features of insulin resistance	6209 (0.6%)	1.50 (1.45, 1.55)
R07.4: Chest pain, unspecified	15 470 (1.5%)	1.47 (1.43, 1.52)
G62.9: Polyneuropathy, unspecified	694 (0.1%)	1.47 (1.41, 1.53)
Z92.2: Personal history of long-term (current) use of other medicaments	2336 (0.2%)	1.47 (1.42, 1.53)
J44.9: Chronic obstructive pulmonary disease, unspecified	523 (0.1%)	1.46 (1.42, 1.51)
Top 10 medications		
Quinapril	46 541 (4.5%)	1.73 (1.68, 1.78)
Varenicline tartrate	26 037 (2.5%)	1.73 (1.69, 1.76)
Nicotine	64 493 (6.3%)	1.68 (1.65, 1.71)
Simvastatin	140 134 (13.7%)	1.66 (1.62, 1.70)
Glyceryl trinitrate	14 227 (1.4%)	1.65 (1.58, 1.72)
Cilazapril	79 241 (7.7%)	1.60 (1.55, 1.64)
Bupropion hydrochloride	25 139 (2.5%)	1.58 (1.54, 1.61)
Tiotropium bromide	3399 (0.3%)	1.52 (1.46, 1.58)
Salbutamol with ipratropium bromide	14 745 (1.4%)	1.46 (1.42, 1.49)
Felodipine	38 670 (3.8%)	1.39 (1.36, 1.43)

a The local hazard ratios for each predictor are adjusted for all other predictors. Values in parentheses are 95% confidence intervals unless otherwise stated.

b Age was centred at the mean value of 49.027. Deprivation quintile was centred around quintile three. The baseline survival estimate at 5 years for the deep learning model, relevant to the mean value of age, deprivation quintile three and the reference group of categorical variables was 0.9812879278038.

c Average and range (in parentheses) of estimated local hazard ratios for all values of the continuous predictor.

The 5-year risk of a first CVD event increased on average by 9% for each 1-year increment in age, for women and men. The effect of age was more conspicuous among people in their sixties and seventies (11% and 13% risk increase in women and men, respectively) than among people in their thirties (6% risk increase for both women and men). Event risk at any time during follow-up was greater among Māori and Pacific women and Māori, Pacific and Indian men but lower among Indian and Other women and Other men compared with their European counterparts. Each increment in quintile of deprivation increased the CVD event risk by 16% in women and 10% in men.

Among the ICD10-AM codes, current tobacco use was associated with a doubling in CVD event risk in women (HR = 2.04, 95% CI: 1.99, 2.10) and an increase of 36% in men (95% CI: 31%, 41%). Codes related to essential hypertension, chest pain, diabetes, general anaesthesia for patients with severe systemic disease, chronic obstructive pulmonary disease, computerized tomography of the brain, history of long-term use of medications, retinopathy and retinal vascular changes, and chronic renal failure were associated with risk increases between 13% and 98% for both women and men. Hospital-recorded alcohol use was associated with CVD event risk in women (45% risk increase, 95% CI: 40%, 50%) but not in men (1% risk increase, 95% CI: −1%, 4%). Some codes were associated with a 7% to 10% decrease in CVD event risk, such as childbirth in women and cycling injuries in men.

People with an increased risk of a CVD event were more likely to have been dispensed smoking cessation medications (nicotine, varenicline tartrate, buproprion hydrochloride), medications used for the treatment of raised blood pressure (cilazapril, furosemide, quinapril, felodipine, glyceryl trinitrate), bronchodilators (salbutamol with ipratropium bromide, tiotropium bromide) and statins (simvastatin). These findings were similar between women and men. Interestingly, dispensing of malathion (a head lice treatment) was also associated with increased CVD event risk in both women (37%, 95% CI: 33%, 41%) and men (32%, 95% CI: 28%, 36%).

### Comparison with Cox proportional hazards models

**Figure 2:**
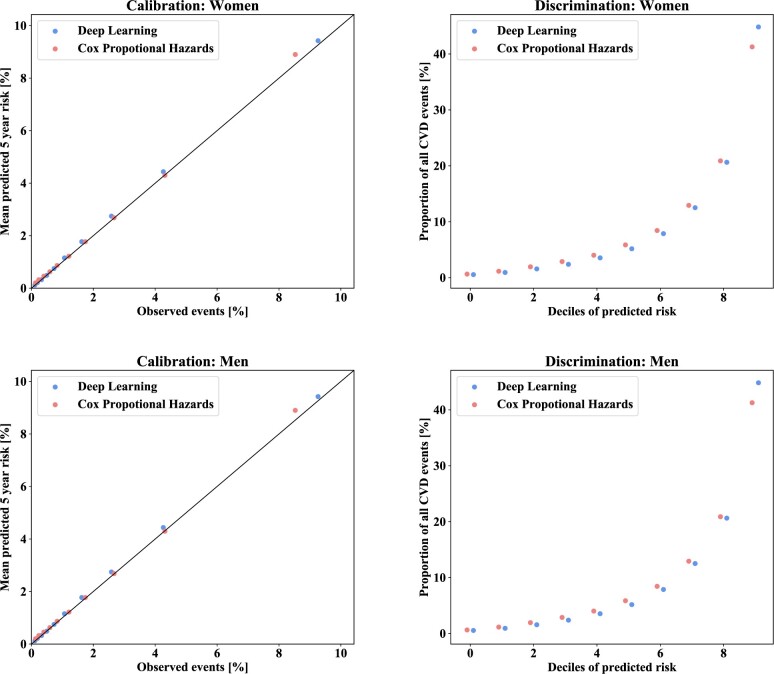
Calibration and discrimination of the deep learning models and Cox proportional hazards models for women and men. The calibration plots show the mean estimated 5-year risk plotted against the proportion of cardiovascular disease events that occurred over 5 years, for deciles of predicted risk. The diagonal line represents perfect calibration. The discrimination plots show the proportion of total observed events that occurred in each decile of predicted risk

**Figure 3:**
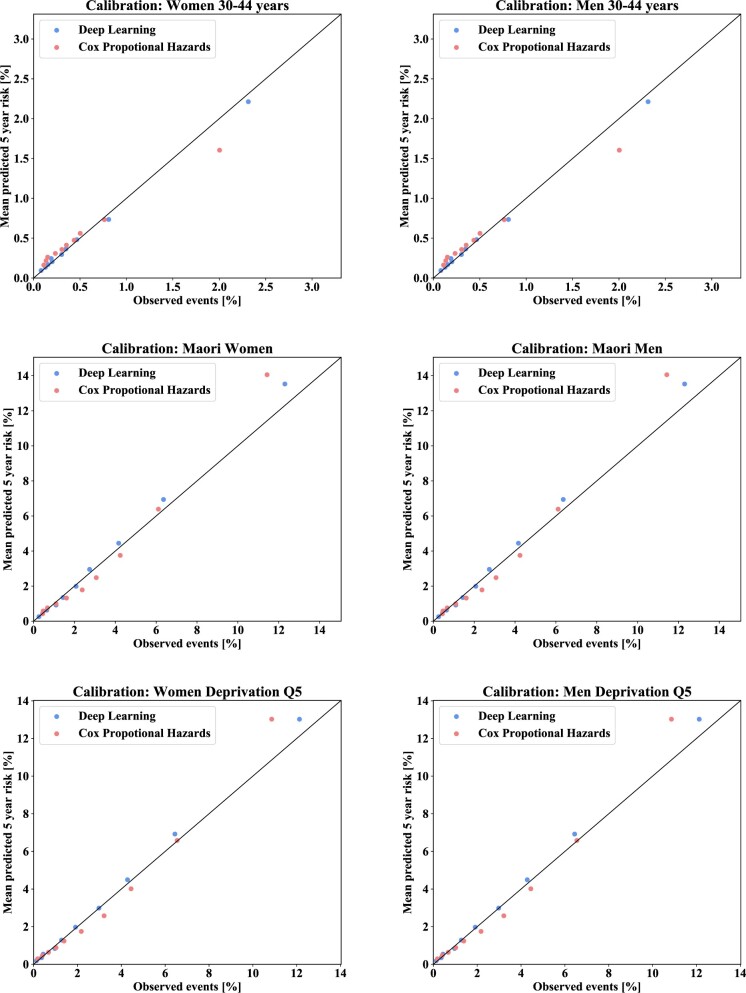
Calibration plots for the deep learning models and Cox proportional hazards models in specific New Zealand sub-populations (women and men aged 30–44 years, Māori women and men and most deprived women and men), suggesting improved calibration for the deep learning models

Both the deep learning models and the traditional Cox models showed good calibration and discrimination, for both women and men (Figure 2). However, the proportion of explained variance was larger for the deep learning models than for Cox models (0.468 vs 0.425 in women and 0.383 vs 0.348 in men, *P *< 0.0001; Table 4). Similarly, discrimination and calibration were better for the deep learning models in terms of Royston and Sauerbrei’s D statistic, Harrell’s C statistic and integrated Brier score, although differences were relatively small (Table 4). A qualitative evaluation of the calibration plots for sub-populations stratified by 15-year age bands, ethnicity, quintiles of deprivation and dispensing of preventive medications also suggested better calibration of the deep learning models (Supplementary Figures S2–S15, available as Supplementary data at *IJE* online). Specific examples of differences in calibration between the two models in women and men aged 30–44 years, Māori women and men and most deprived women and men are shown in Figure 3. Overall, performance metrics associated with the models for women were better than those for men (Table 4).

**Table 4:** Performance metrics for the deep learning models and traditional Cox proportional hazards models

Performance metric	Statistic (95% CI)					
	Women			Men		
	Deep learning	Cox proportional hazards	*P*-value^d^	Deep learning	Cox proportional hazards	*P*-value^d^
R^2^^a^	**0.468 (0.465, 0.471)**	0.425 (0.423, 0.428)	<0.0001	**0.383 (0.381, 0.385)**	0.348 (0.346, 0.350)	<0.0001
D statistic^b^	**1.92 (1.91, 1.93)**	1.76 (1.75, 1.77)	<0.0001	**1.61 (1.60, 1.62)**	1.49 (1.49, 1.50)	<0.0001
Harrell’s C^b^	**0.813 (0.812, 0.814)**	0.795 (0.794, 0.797)	<0.0001	**0.771 (0.771, 0.772)**	0.759 (0.758, 0.759)	<0.0001
Integrated Brier score^c^	**0.00971 (0.00970, 0.00972)**	0.00978 (0.00977, 0.00979)	<0.0001	**0.0176 (0.0176, 0.0176)**	0.0177 (0.0177, 0.0177)	<0.0001

Better results are in bold. 95% confidence intervals (CIs) are computed using 5 × 2 cross-validation.

a Royston and Sauerbrei’s R^2^ measures how much of the time-to-event occurring is explained by the model. Higher values indicate that more variation is accounted for by the model.^16^

b Royston and Sauerbrei’s D statistic and Harrell’s C statistic are measures of discrimination. Better discrimination is indicated by higher values.

c The integrated Brier score is affected by both calibration and discrimination.^18^^,^^19^ Better calibration and discrimination are indicated by lower values.

d Computed using combined 5 × 2 F tests.

Hazard ratios determined by the traditional Cox models were comparable in magnitude to the local HRs determined by the deep learning models (Table 5), although slightly smaller for ethnic groups and larger for history of diabetes and atrial fibrillation and baseline dispensing of medications. Coefficients of the corresponding CVD risk equations are reported in Supplementary Table S8, available as Supplementary data at *IJE* online.

**Table 5:** Adjusted hazard ratios for time to cardiovascular disease event within 5 years, determined by the Cox proportional hazards models

Predictors	Adjusted hazard ratios (95% CI)^a^	
	Women	Men
Age (per year)^b^	1.09 (1.09, 1.09)	1.08 (1.08, 1.08)
Ethnicity		
European	1	1
Māori	1.84 (1.78, 1.91)	1.55 (1.51, 1.61)
Pacific	1.40 (1.33, 1.48)	1.26 (1.20, 1.32)
Indian	0.910 (0.837, 0.989)	1.18 (1.11, 1.25)
Other	0.688 (0.647, 0.732)	0.751 (0.717, 0.787)
Deprivation quintile (per quintile)^b^	1.15 (1.14, 1.16)	1.11 (1.10, 1.12)
Diabetes	2.43 (2.26, 2.62)	2.20 (2.07, 2.33)
Atrial fibrillation	2.54 (2.14, 3.01)	1.99 (1.80, 2.20)
Medications dispensed at baseline		
Blood-pressure-lowering	2.24 (2.13, 2.35)	1.86 (1.79, 1.94)
Lipid-lowering	1.02 (0.956, 1.08)	0.942 (0.903, 0.982)
Antiplatelet/anticoagulant	1.48 (1.42, 1.55)	1.32 (1.27, 1.37)
Interactions		
Age (years)*blood pressure-lowering-medication	0.975 (0.972, 0.978)	0.976 (0.974, 0.979)
Age (years)*diabetes	0.983 (0.980, 0.987)	0.982 (0.979, 0.985)
Age (years)*atrial fibrillation	0.984 (0.975, 0.994)	0.985 (0.979, 0.991)
Blood pressure-lowering-medication*diabetes	0.878 (0.807, 0.956)	0.858 (0.803, 0.917)
Antiplatelet/anticoagulant medications*diabetes	0.804 (0.744, 0.868)	0.855 (0.803, 0.910)
Blood-pressure-lowering medication*lipid-lowering medication	0.858 (0.797, 0.923)	0.941 (0.892, 0.994)

a The hazard ratios for each predictor are adjusted for all other predictors.

b Age was centred in women and men separately using their mean values. For age, the mean value in women was 49.021 and the mean value in men was 49.027. Deprivation quintile was centred around quintile three in women and men. The baseline survival estimate at 5 years relevant to the mean value of age, deprivation quintile three and the reference group of categorical variables was 0.9905071151673 among women and 0.9782399916755 among men.

### Neural network ablation study

When comparing the deep learning models developed with and without redundant predictors, the most evident difference was that the models without pre-specified predictors for diabetes status and previous hospitalization for atrial fibrillation associated a higher risk of a CVD event with ICD10-AM codes for these conditions (Supplementary Tables S9 and S10, available as Supplementary data at *IJE* online). Performance measures were slightly better for the deep learning models with redundant predictors (e.g. the proportion of explained variance was 0.468 vs 0.461 in women and 0.383 vs 0.379 in men; Supplementary Table S11, available as Supplementary data at *IJE* online).

## Discussion

This study developed deep learning models to predict the 5-year risk of a fatal or non-fatal CVD event across the entire primary prevention population of New Zealand, using only predictors available in routinely collected administrative health data. The new models account for censoring of unobserved events and were used to gain insight into diagnoses, procedures and medications associated with increased risk of CVD events.

Compared with traditional Cox proportional hazards models, the deep learning models showed improved calibration and discrimination across the whole population and in sub-populations stratified by 15-year age bands, ethnicity, quintiles of deprivation and dispensing of preventive medications. Inclusion of pre-specified predictors and interaction terms facilitated comparison between the deep learning and Cox models and allowed direct estimation of the gain in predictive performance achieved by the deep learning extension to traditional models. However, these pre-specified terms were partly redundant and might have biased some of the estimated local hazard ratios towards one. Model performance degraded slightly when these terms were removed, likely because the pre-specified predictors for diabetes status (derived from the national Virtual Diabetes Registry) and previous hospitalization for atrial fibrillation (in the 20 years before the index date) capture a larger proportion of people with these conditions than the ICD10-AM codes associated with hospital admissions in the 5 years prior to the index date. In addition, the deep learning models that included first-order interaction terms between pre-specified predictors as part of the input might have been able to compute relevant higher-order interaction terms, improving their predictive performance. Since the deep learning models adjusted for any diagnoses, procedures and medications in the 5 years prior to the index date, the estimated local hazard ratios might also have been affected by collider bias and multicollinearity. Accordingly, the local hazard ratios serve primarily to demonstrate the face validity and biological plausibility of the deep learning models rather than being interpretable as estimates of causal effects.

A few previous studies investigated the use of machine learning for CVD risk prediction using large-scale data from prospective study cohorts,^20^^,^^21^ family practices in the UK,^22^ primary health care centres in Spain^23^ and hospitals and community health service centres in China^24^ and the United States.^25^ They suggest that machine learning improves CVD risk prediction, in agreement with the present findings, although their measures of performance were limited. Moreover, only one of these studies was able to account for censored data through the use of random survival forests.^20^ A recent study comparing machine learning and traditional survival models for CVD risk prediction using UK family practice data shows that machine learning models that ignore censoring produce biased risk estimates, and suggests that survival models that consider censoring and that are explainable, are preferable.^26^ However, the latter study did not evaluate any machine learning models that account for censoring. The present study is the first to use deep learning extensions of survival analysis models for CVD risk prediction, using routinely collected health data for a national population.

Previous studies generally used random forests to rank the importance of predictors, an approach which might not always be reliable due to bias towards inclusion of predictors with many split points.^27^^,^^28^ In the present study, the associations of individual ICD10-AM codes and medications with the outcome were described using estimated hazard ratios. The most relevant diagnoses (e.g. current tobacco use, essential hypertension, chest pain, diabetes, chronic obstructive pulmonary disease, history of long-term use of medications, retinopathy and retinal vascular changes) and medications (related to smoking cessation, treatment of raised blood pressure and heart failure, bronchodilators and statins) generally aligned with current knowledge about CVD risk predictors. The results also support previous findings regarding sex-related differences in cardiovascular risk predictors, such as the more deleterious effect of smoking in women and the particularly high risk associated with significant renal disease in men.^29^

In conclusion, the proposed deep learning extensions of survival analysis models enabled 5-year CVD risk predictions for the primary prevention population of New Zealand with improved calibration and discrimination. The developed models are freely available and could similarly be used for CVD risk prediction and population health planning in other countries where large administrative health datasets can be linked at the individual person level. For example, they may be used to estimate future CVD incidence, identify target sub-populations for prevention and assess the likely benefit of health policies and interventions in different risk groups. The proposed method to compute local hazard ratios has applications beyond CVD risk prediction and could be used in time-to-event analyses to identify diagnoses, procedures and medications associated with other conditions.

Further improvements to predictive accuracy through additional data sources such as laboratory tests, and the development of frameworks which integrate machine learning and causal inference (e.g. through the use of causal regularizers which steer deep learning models towards causally interpretable solutions)^30^ represent interesting avenues for future research.

## Data availability

All data were obtained from the New Zealand Ministry of Health. Access to the dataset can be requested from R.J. at [rt.jackson@auckland.ac.nz]. Requests will be granted after consideration by the VIEW research programme Governance Group, agreement by the New Zealand Ministry of Health and ethical approval by the New Zealand Multi-Region Ethics Committee. The developed algorithms, trained deep learning models and tabulated results are publicly available at [https://github.com/VIEW2020/Varianz2012].

## Supplementary data

Supplementary data are available at *IJE* online.

## Funding

This work was supported by the Health Research Council of New Zealand (grant numbers 11/800, 14/010 to S.M.), S.M. was supported by a New Zealand Health Research Council Clinical Research Training Fellowship, C.B. is supported by National Drug and Alcohol Research Centre (NDARC) and University of New South Wales Scientia PhD Scholarships, and K.P. is supported by a New Zealand Heart Foundation Hynds Senior Fellowship. The study funders/sponsors had no role in the study design, collection, analysis or interpretation of data.

## Author contributions

S.B., K.P., L.J., R.J. designed the study. B.W. performed data cleaning and pre-processing. S.M. implemented the Cox proportional hazards models. S.B. implemented the deep learning models and drafted the manuscript. S.B. and C.B. evaluated the performance of the Cox and deep learning models. All authors contributed to the interpretation of findings and manuscript revisions.

## Conflict of interest

No financial or other relationships with any organizations that might have an interest in the submitted work in the previous 3 years.

## Supplementary Material

dyab258_Supplementary_Data
