# Supplementary material for: Predicting cardiovascular risk from national administrative databases using a combined survival analysis and deep learning approach
Source: Int J Epidemiol. 2021 Dec 15;51(3):931–44. doi: 10.1093/ije/dyab258 (PMC9189958; doi:10.1093/ije/dyab258)
Supplement: dyab258_Supplementary_Data [file dyab258_supplementary_data.pdf]

Predicting cardiovascular risk from national administrative databases using a combined survival analysis and deep learning approach

SUPPLEMENTARY MATERIAL

SUPPLEMENTARY METHODS

ICD10-AM codes used to classify prior history of CVD and heart failure
The International Statistical Classification of Diseases and Related Health Problems, Tenth Revision, Australian Modification (ICD-10-AM) codes[1] used to classify prior history of CVD and heart failure are listed in Supplementary Table S1. In addition, medication dispensing records were used to augment identification of pre-existing ischaemic heart disease and heart failure. Any person with three or more dispensing episodes of an anti-anginal medication (as a proxy for ischaemic heart disease) during the previous five years was excluded from the dataset. Any person with three or more dispensing episodes of a loop diuretic during the previous five years was considered to have pre-existing heart failure and also excluded from the dataset. The medications are listed in Supplementary Table S2. Individuals with metolazone dispensing (another heart failure medication) at least once in the six month period prior to the index date would also have been excluded, but none were identified in the dataset.

Blood pressure lowering medication
A person was considered to be treated with blood pressure lowering medication at baseline if they had been dispensed a blood pressure lowering medication during the latter six months of 2012 (the baseline year for this study). Included blood pressure lowering medications are listed in Supplementary Table S3.

Lipid lowering medication
A person was considered to be treated with lipid lowering medication at baseline if they had been dispensed a lipid lowering medication during the latter six months of 2012 (the baseline year for this study). Lipid lowering medications are listed in Supplementary Table S4.

Antiplatelet/anticoagulant medication
A person was considered to be treated with antiplatelet or anticoagulant medication at baseline if they had been dispensed an antiplatelet or anticoagulant medication during the latter six months of 2012 (the baseline year for this study). Included antiplatelet and anticoagulant medications are listed in Supplementary Table S5.

Non-fatal CVD event
A CVD event was defined as a hospitalisation with a discharge diagnosis code consistent with CVD or heart failure. The categories of CVD and included codes are listed in Supplementary Table S6. The date of admission was used to define the date of a CVD hospitalisation event.

Fatal CVD event
A death was classified as a CVD death if the underlying cause in the mortality dataset was listed in Supplementary Table S6. The underlying cause of death was taken from the mortality dataset. Coding of the underlying cause of death in the New Zealand mortality collection is assigned according to standardised rules set out in the World Health Organization ICD Rules and Guidelines for Mortality Coding[1] using documentation from the death certificate.
Additionally, any death that occurred within 28 days of a hospitalisation associated with a discharge diagnosis of any CVD event was classified as a CVD death, regardless of the underlying cause of death in the mortality dataset.

Date of death
Date of death was obtained from the mortality collection dataset and comes from the official death certificate.

Hyperparameter optimisation for the deep learning models
The optimised neural network hyperparameters included whether to pass time as an additional covariate to the relative risk function (to account for non-proportional hazards),[2] the embedding dimension, the use of GRUs[3] or long short-term memory (LSTMs)[4] as gating mechanisms, the number of stacked recurrent neural network layers and the percentage of dropout[5] during training, the number of fully-connect layers, whether to add embeddings describing the type of code, whether to ignore, concatenate or add as embeddings the time difference between codes, how to summarise the output of the recurrent layers (use of the last hidden state or max/sum/average/attention pooling[6] of outputs), and the learning rate of the Adam optimiser. Further details are reported in Supplementary Table S7.
Optimisation was performed using a Tree-structured Parzen Estimator (TPE)[7] over 100 trials, where the value of a trial corresponded to the loss on the validation data and unsuccessful trials were pruned using successive halving.[8] Early-stopping was implemented by terminating training if the loss function computed on the validation data did not improve over ten consecutive iterations.

The optimisation process was iterated three times for both sex-specific models, with the aim of obtaining a common set of hyperparameters. Following each iteration, hyperparameters selected for both sex-specific models were fixed to focus the search on the remaining hyperparameter space. Final hyperparameter differences between the two models were settled heuristically by choosing the more expressive or more common settings. Repeated training using the final set of hyperparameters minimised the loss on the validation data for both sex-specific models.

SUPPLEMENTARY TABLES

**Category**	**ICD10-AM Codes**
Cardiac arrest	I46†
Ischaemic heart disease	I20†, I21†, I22†, I23†, I24†(except I241, Dressler syndrome), I25†
Coronary procedure	3530400-3530401, 3530500-3530501, 3530906-3530909, 3531000-3531005, 3849700-3849707, 3850000-3850004, 3850300-3850304, 9020100-9020103. 3845619, 3850500, 3850700, 3850800, 3850900, 3863700, Z951, Z955, Z958, Z959
Ischaemic stroke	I63†, I64, I693, I694
Haemorrhagic stroke	I60†, I61†, I690, I691
Other cerebrovascular disease	G45† (except G454, transient global amnesia), I65†, I66†, I670, I672, I698
Peripheral vascular disease	I702†, I700, I701, I7020, I708, I709, I71†, I739, I74†, E105†, E115†m E145†
Peripheral vascular disease procedure	327000-3271011, 3270300, 3310000, 3350000, 3270800-3270803, 3311200, 3311500, 3311800, 3312100, 3315100, 3315400, 3315700, 3316000, 3350900, 3351200, 3351500, 3271200-3271201, 3271500-3271503, 3271800-3271801, 3273900, 3274200, 3274500, 3274800, 3275100-3275103, 3275400-3275402, 3275700-3275701, 3351501, 3352100, 3354200, 3273000-3273001, 3273300-3273301, 3273600, 3353001, 3353300, 3353600, 3276300-3276303, 3276305-3276314, 3276316-3276319, 3305000, 3305500, 3307500, 3308000, 3312400, 3312700, 3313000, 3316300, 3317800, 3318100, 3350600-3350601, 3351800, 3352400, 3352700,
Congestive heart failure	I110, I130, I132, I50, I500, I501, I509

Supplementary Table S1. ICD10-AM codes used to classify prior history of CVD and heart failure. † Denotes that all subcategories were included, unless specifically noted.

**Class**	**Medications**
Loop diuretic	Frusemide, Amiloride with frusemide, Bumetanide
Anti-anginal	Glyceryl trinitrate, Isosorbide mononitrate, Isosorbide dinitrate, Nicorandil, Pentaerythritol tetranitrate, Perhexiline

Supplementary Table S2. Loop diuretic and anti-anginal medication definitions.

**Class**	**Medications**
Angiotensin converting enzyme (ACE) inhibitors	Captopril, Perindopril, Lisinopril, Benazepril, Quinapril, Cilazapril, Enalapril maleate, Trandolapril, Quinapril with hydrochlorothiazide, Captopril with hydrochlorothiazide, Lisinopril with hydrochlorothiazide, Enalapril maleate with hydrochlorothiazide, Cilazapril with hydrochlorothiazide
Angiotensin II receptor blockers	Losartan with hydrochlorothiazide, Candesartan cilexetil, Losartan potassium, Losartan with hydrochlorothiazide, Losartan potassium with hydrochlorothiazide, Losartan
Beta-blockers	Carvedilol, Celiprolol, Timolol, Sotalol, Propranolol, Pindolol, Oxprenolol, Nadolol, Metoprolol tartrate, Metoprolol succinate, Labetalol, Atenolol, Alprenolol, Acebutolol, Acebutolol with hydrochlorothiazide, Pindolol with clopamide, Atenolol with chlorthalidone, Bisoprolol fumarate
Calcium channel blockers	Amlodipine, Diltiazem hydrochloride, Felodipine, Isradipine, Nifedipine, Verapamil hydrochloride, Verapamil Hydrochloride

Thiazide diuretics	Cilazapril with hydrochlorothiazide, Cyclopenthiazide, Enalapril maleate with hydrochlorothiazide, Indapamide, Lisinopril with hydrochlorothiazide, Losartan, Losartan potassium with hydrochlorothiazide, Losartan with hydrochlorothiazide, Losartan with Hydrochlorothiazide, Methyclothiazide, Methyldopa with hydrochlorothiazide, Quinapril with hydrochlorothiazide, Triamterene with hydrochlorothiazide
Other	Amiloride hydrochloride, Amiloride hydrochloride with furosemide, Amiloride hydrochloride with hydrochlorothiazide, Clonidine, Clonidine hydrochloride, Clonidine Hydrochloride, Hydralazine hydrochloride, Methyldopa, Methyldopa with hydrochlorothiazide, Pindolol with clopamide, Triamterene with hydrochlorothiazide

Supplementary Table S3. Blood pressure lowering medication classification.

**Class**	**Medications**
Statin	Pravastatin, Simvastatin, Atorvastatin, Fluvastatin, Ezetimibe with simvastatin
Other lipid lowering medication	Acipimox, Bezafibrate, Cholestyramine, Clofibrate, Colestipol hydrochloride, Ezetimibe, Ezetimibe with simvastatin, Gemfibrozil, Nicotinic acid

Supplementary Table S4. Lipid lowering medication classification.

**Class**	**Medications**
Antiplatelet	Aspirin, Clopidogrel, Ticagrelor, Dipyridamole, Prasugrel, Ticlopidine hydrochloride
Anticoagulant	Warfarin, Dabigatran, Phenindione, Rivaroxaban

Supplementary Table S5. Antiplatelet and anticoagulant medication classification.

**Category**	**ICD10-AM Codes**
Cardiac arrest	I46†
Ischaemic heart disease	I20†, I21†, I22†, I23†, I24†(except I241, Dressler syndrome), I253-I256
Coronary procedure	3530400, 3530500, 3530906-3530909, 3531000-3531002, 3849700-3849707, 3850000-3850004, 3850300-3850304, 9020100-9020103. 3845619, 3863700, Z958, Z959
Ischaemic stroke	I63†, I64
Haemorrhagic stroke	I60†, I61†, I690, I691
Other cerebrovascular disease	G45† (except G454, transient global amnesia), I65†, I66†, I670, I672
Peripheral vascular disease	I702†, I700, I701, I7020, I708, I709, I71†, I739, I74†, E105†, E115†, E145†
Peripheral vascular disease procedure	327000-3271011, 3270300, 3310000, 3350000, 3270800-3270803, 3311200, 3311500, 3311800, 3312100, 3315100, 3315400, 3315700, 3316000, 3350900, 3351200, 3351500, 3271200-3271201, 3271500-3271503, 3271800-3271801, 3273900, 3274200, 3274500, 3274800, 3275100-3275103, 3275400-3275402, 3275700-3275701, 3351501, 3352100, 3354200, 3273000-3273001, 3273300-3273301, 3273600, 3353001, 3353300, 3353600, 3276300-3276303, 3276305-3276314, 3276316-3276319, 3305000, 3305500, 3307500, 3308000, 3312400, 3312700, 3313000, 3316300, 3317800, 3318100, 3350600-3350601, 3351800, 3352400, 3352700,
Congestive heart failure	I110, I130, I132, I50, I500, I501, I509

Supplementary Table S6. ICD10-AM codes used to classify fatal and nonfatal CVD events. † Denotes that all subcategories were included, unless specifically noted.

**Hyperparameter**	**Values**
Nonproportional hazards (by passing time as an additional covariate to the network)[2]	True, **False**
Embedding dimension	16, **32**, 64, 128
Recurrent layer type	**GRU**, LSTM
Number of recurrent layers	1, 2, **3**
Dropout in recurrent layers	0, **0.1**, 0.2, 0.3, 0.4, 0.5
Number of size-preserving fully-connected layers	0, **1**, 2
Add type of code embeddings (primary diagnosis, secondary diagnosis, external cause of injury, or procedure or operation)	**True**, False
Treatment of time difference between codes	Ignore, **concatenate**, add as embeddings
Summation of the outputs of recurrent layers	Last hidden state, max-pooling of outputs, sum of outputs, average-pooling of outputs, **attention-pooling of outputs**
Adam's learning rate	1e-4, **1e-3**, 1e-2

Supplementary Table S7. Optimised deep learning hyperparameters. Selected values are in bold.

Predictors	Coefficient	
	Women	Men
**Age** (per year)[a]	0.0834246	0.0769116
**Ethnicity**		
European	Referent	Referent
Māori	0.6113605	0.4431610
Pacific	0.3387812	0.2305849
Indian	-0.0944792	0.1655833
Other	-0.3739487	-0.2865808
**Deprivation quintile** (per quintile)[b]	0.1392213	0.1057541
**Diabetes**	0.8875899	0.7880961
**Atrial fibrillation**	0.9328362	0.6878545
**Medications dispensed at baseline**		
Blood pressure lowering	0.8056196	0.6218469
Lipid lowering	0.0159035	-0.0600578
Antiplatelet/anticoagulant	0.3926647	0.2782693
**Interactions**		
Age (years)*blood pressure lowering medication	-0.0249163	-0.0239351
Age (years)*diabetes	-0.0169612	-0.0186164
Age (years)*atrial fibrillation	-0.0156994	-0.0154908
Blood pressure lowering medication*diabetes	-0.1297733	-0.1527890
Antiplatelet/anticoagulant medications*diabetes	-0.2184231	-0.1572052
Blood pressure lowering medication*lipid lowering medication	-0.1531903	-0.0604127

Supplementary Table S8. Sex-specific Cox proportional hazards model coefficients and calculation of the absolute risk scores.

[a]Age was centred (mean value in women=49.021; mean value in men=49.027)

[b]Deprivation quintile was centred (quintile 3 in women and men)

RISK SCORES: βx is calculated by multiplying each relevant predictor with the associated beta coefficient. Therefore, 5-year CVD risk = $1 - \text{Baseline survival}^{\exp(\beta x)} *100$

<u>Risk score for WOMEN</u>: $1 - 0.9905071151673^{\exp(\beta x)} *100$

<u>Risk score for MEN</u>: $1 - 0.9782300016755^{\exp(\beta x)} *100$

**Women (N = 1 141 925)**		**Deep learning model**
**Predictors**	**N (%)**	**Adjusted local HRs (95% CI)**[a]
**Age** (per year)[b]		1.09 (1.06, 1.11)[c]
**Ethnicity**		
European	797 571 (69.8%)	1
Māori	132 802 (11.6%)	1.98 (1.97, 1.99)
Pacific	60 965 (5.3%)	1.68 (1.67, 1.69)
Indian	38 481 (3.4%)	0.928 (0.921, 0.935)
Other	112 106 (9.8%)	0.721 (0.717, 0.725)
**Deprivation quintile** (per quintile)[b]		1.16 (1.15, 1.16)[c]
**Top-10 Diagnoses and Procedures**		
I48: Atrial fibrillation and flutter	4137 (0.4%)	1.68 (1.61, 1.74)
Z72.0: Tobacco use, current	84 589 (7.4%)	1.67 (1.61, 1.74)
E11.8: Type 2 diabetes mellitus with unspecified complication	2189 (0.2%)	1.66 (1.59, 1.72)
Z92.22: Personal history of long-term (current) use of other medicaments, insulin	2169 (0.2%)	1.64 (1.58, 1.70)
E11.71: Non-insulin-dependent diabetes mellitus with multiple complications, stated as uncontrolled	643 (0.1%)	1.62 (1.55, 1.69)
92514-39: General anaesthesia, ASA 3 (Patient with severe systemic disease that limits activity), nonemergency or not known	10 961 (1.0%)	1.58 (1.51, 1.65)
E11.72: Type 2 diabetes mellitus with features of insulin resistance	7220 (0.6%)	1.57 (1.51, 1.63)
R07.3: Other chest pain	9222 (0.8%)	1.55 (1.48, 1.61)
E66.9: Obesity, unspecified	4058 (0.4%)	1.52 (1.46, 1.57)
E11.9: Type 2 diabetes mellitus without complication	3334 (0.3%)	1.52 (1.46, 1.57)
**Top-10 Medications**		
Nicotine	79 506 (7.0%)	1.67 (1.63, 1.72)
Cilazapril	76 762 (6.7%)	1.57 (1.53, 1.61)
Furosemide [Frusemide]	13 340 (1.2%)	1.50 (1.45, 1.55)
Varenicline tartrate	31 750 (2.8%)	1.50 (1.46, 1.53)
Warfarin sodium	7988 (0.7%)	1.47 (1.42, 1.52)
Quinapril	48 373 (4.2%)	1.45 (1.42, 1.48)
Tiotropium bromide	4078 (0.4%)	1.44 (1.39, 1.49)
Aspirin	117 380 (10.3%)	1.43 (1.40, 1.45)
Malathion	22 441 (2.0%)	1.39 (1.35, 1.43)
Bupropion hydrochloride	30 796 (2.7%)	1.38 (1.35, 1.42)

Supplementary Table S9. Adjusted local hazard ratios (HRs) for time to CVD event within five years for women, determined by the deep learning model without redundant predictors (only the 10 diagnoses and procedures, and the 10 medications, associated with the largest hazard ratios are reported). CI: confidence interval.

[a]The local hazard ratios for each predictor are adjusted for all other predictors. Values in parentheses are 95% confidence intervals unless otherwise stated.

[b]Age was centred at the mean value of 49.021. Deprivation quintile was centred around quintile three. The baseline survival estimate at five years for the deep learning model, relevant to the mean value of age, deprivation quintile three and the reference group of categorical variables was 0.9927233213429.

[c]Average and range (in parentheses) of estimated local hazard ratios for all values of the continuous predictor.

Men (N = 1 022 947)		Deep learning model
**Predictors**	**N (%)**	**Adjusted local HRs (95% CI)**[a]
**Age** (per year)[b]		1.09 (1.06, 1.13)[c]
**Ethnicity**		
European	734 891 (71.8%)	1
Māori	106 912 (10.5%)	1.70 (1.69, 1.70)
Pacific	54 659 (5.3%)	1.43 (1.42, 1.44)
Indian	36 248 (3.5%)	1.39 (1.38, 1.40)
Other	90 237 (8.8%)	0.786 (0.783, 0.790)
**Deprivation quintile** (per quintile)[b]		1.10 (1.09, 1.10)[c]
**Top-10 Diagnoses and Procedures**		
I10: Essential (primary) hypertension	12 604 (1.2%)	2.46 (2.37, 2.56)
E11.65: Type 2 diabetes mellitus with poor control	1792 (0.2%)	1.95 (1.87, 2.04)
I48: Atrial fibrillation and flutter	7025 (0.7%)	1.83 (1.76, 1.91)
J44.0: Chronic obstructive pulmonary disease with acute lower respiratory infection	1529 (0.1%)	1.70 (1.63, 1.78)
E11.71: Non-insulin-dependent diabetes mellitus with multiple complications, stated as uncontrolled	663 (0.1%)	1.68 (1.60, 1.76)
Z92.1: Personal history of long-term (current) use of anticoagulants	1524 (0.1%)	1.64 (1.57, 1.71)
E11.72: Type 2 diabetes mellitus with features of insulin resistance	6209 (0.6%)	1.63 (1.56, 1.71)
E66.8: Other obesity	1261 (0.1%)	1.62 (1.55, 1.69)
Z92.22: Personal history of long-term (current) use of other medicaments, insulin	1928 (0.2%)	1.62 (1.55, 1.69)
G62.9: Polyneuropathy, unspecified	694 (0.1%)	1.58 (1.52, 1.65)
**Top-10 Medications**		
Quinapril	46 541 (4.5%)	1.90 (1.84, 1.96)
Cilazapril	79 241 (7.7%)	1.79 (1.75, 1.83)
Varenicline tartrate	26 037 (2.5%)	1.69 (1.65, 1.73)
Simvastatin	140 134 (13.7%)	1.68 (1.65, 1.72)
Nicotine	64 493 (6.3%)	1.68 (1.65, 1.72)
Glyceryl trinitrate	14 227 (1.4%)	1.68 (1.60, 1.75)
Metformin hydrochloride	48 389 (4.7%)	1.62 (1.57, 1.68)
Tiotropium bromide	3399 (0.3%)	1.56 (1.51, 1.62)
Warfarin sodium	12 201 (1.2%)	1.56 (1.52, 1.59)
Bupropion hydrochloride	25 139 (2.5%)	1.53 (1.50, 1.56)

Supplementary Table S10. Adjusted local hazard ratios (HRs) for time to CVD event within five years for men, determined by the deep learning model without redundant predictors (only the 10 diagnoses and procedures, and the 10 medications, associated with the largest hazard ratios are reported). CI: confidence interval.

[a]The local hazard ratios for each predictor are adjusted for all other predictors. Values in parentheses are 95% confidence intervals unless otherwise stated.

[b]Age was centred at the mean value of 49.027. Deprivation quintile was centred around quintile three. The baseline survival estimate at five years for the deep learning model, relevant to the mean value of age, deprivation quintile three and the reference group of categorical variables was 0.9828069641498.

[c]Average and range (in parentheses) of estimated local hazard ratios for all values of the continuous predictor.

**Performance metric**	**Statistic (95% CI)**					
	**Women**			**Men**		
	**Deep learning with redundant predictors**	**Deep learning without redundant predictors**	***p*-value[d]**	**Deep learning with redundant predictors**	**Deep learning without redundant predictors**	***p*-value[d]**
R-squared[a]	**0.468 (0.465, 0.471)**	0.461 (0.458, 0.464)	0.02	**0.383 (0.381, 0.385)**	0.379 (0.378, 0.381)	0.02
D statistic[b]	**1.92 (1.91, 1.93)**	1.89 (1.88, 1.91)	0.02	**1.61 (1.60, 1.62)**	1.60 (1.59, 1.61)	0.02
Harrell's C[b]	**0.813 (0.812, 0.814)**	0.811 (0.810, 0.813)	0.06	**0.771 (0.771, 0.772)**	0.771 (0.770, 0.771)	0.08
Integrated Brier score[c]	**0.00971 (0.00970, 0.00972)**	0.00973 (0.00971, 0.00974)	0.01	**0.0176 (0.0176, 0.0176)**	0.0176 (0.0176, 0.0176)	0.03

Supplementary Table S11. Performance metrics for the deep learning models with and without redundant predictors. Better results are in bold. 95% confidence intervals (CIs) are computed using 5x2 cross validation.

[a]Royston and Sauerbrei's R-squared measures how much of the time-to-event occurring is explained by the model. Higher values indicate that more variation is accounted for by the model.[9]

[b]Royston and Sauerbrei's D statistic and Harrell's C statistic are measures of discrimination. Better discrimination is indicated by higher values. Royston and Sauerbrei's D statistic represents the log hazard ratio of two equally sized prognostic groups identified by dividing the study population according to the median of the prognostic index. Therefore, the D statistic quantifies the prognostic separation of survival curves between these two groups.[9] Harrell's C statistic estimates the proportion of pairs of individuals where concordance is observed between predictions and outcomes.[10]

[c]The expected Brier score may be interpreted as the mean square error of prediction, and is affected by both calibration and discrimination.[11,12] Better discrimination and calibration is indicated by lower values. The integrated Brier score averages model performance over all available times.

[d]Computed using combined 5x2 *F* tests.[13]

SUPPLEMENTARY FIGURES

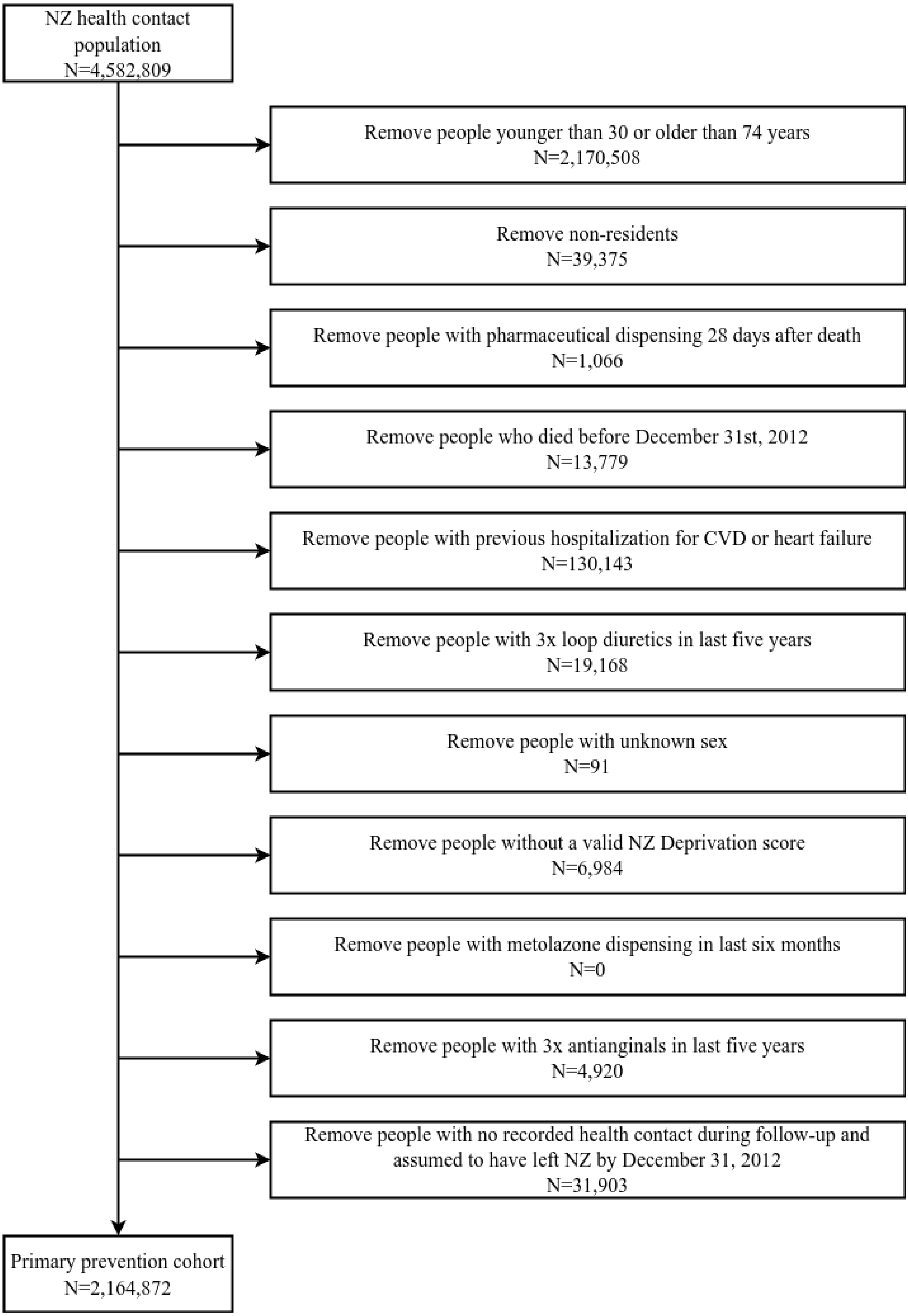

Supplementary Figure S1. Cohort development flowchart.

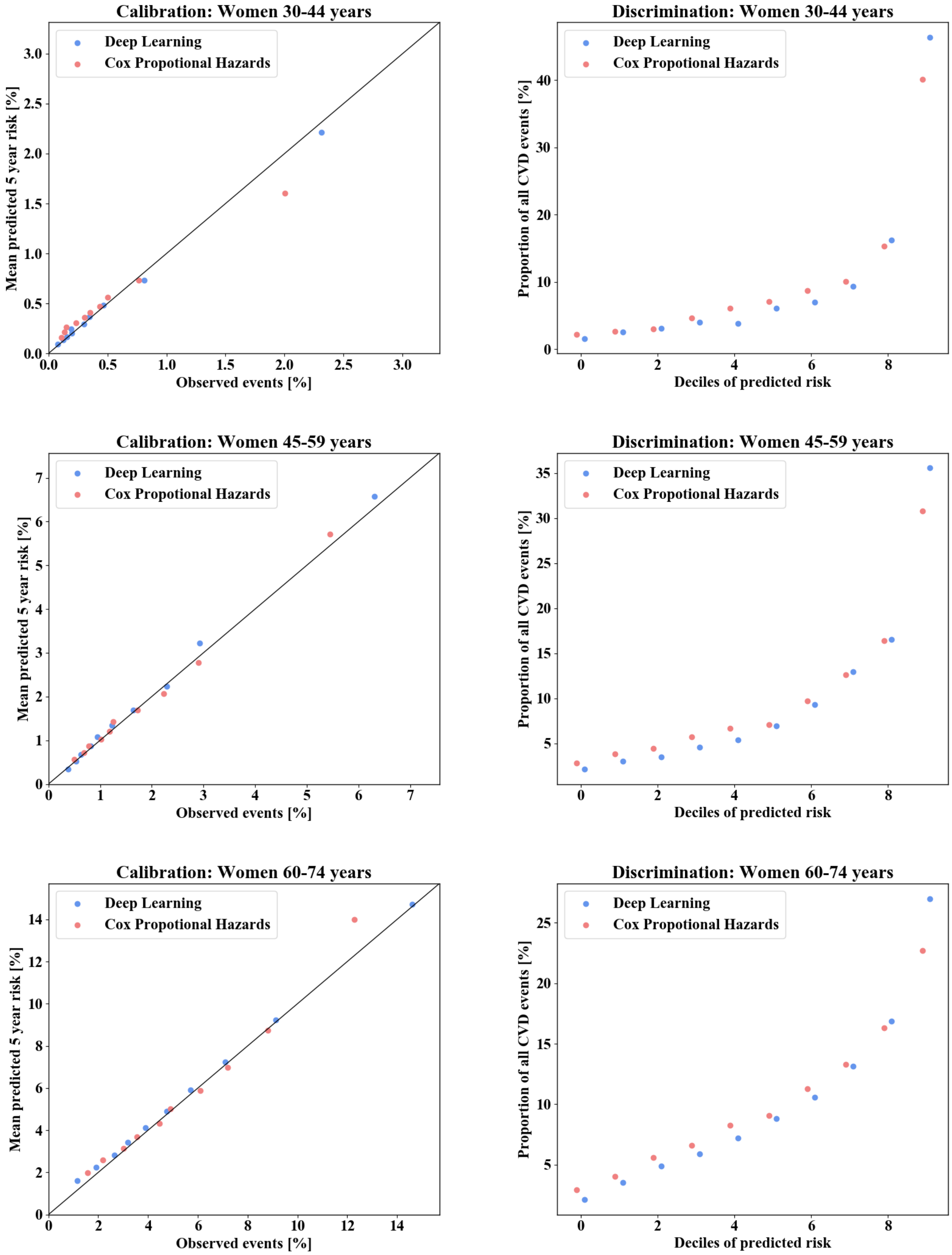

Supplementary Figure S2. Calibration and discrimination in women stratified by 15-year age bands.

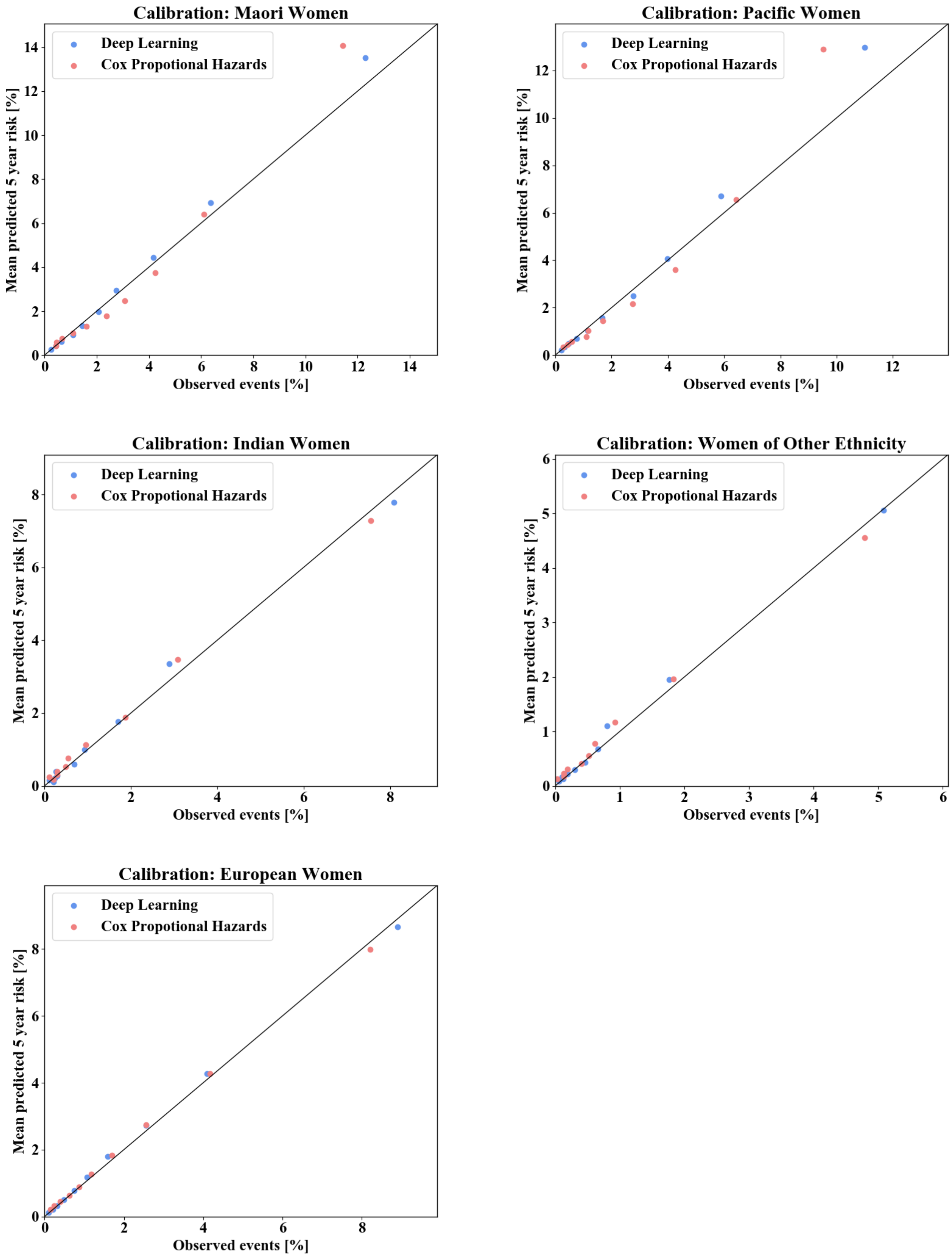

Supplementary Figure S3. Calibration in women stratified by ethnicity.

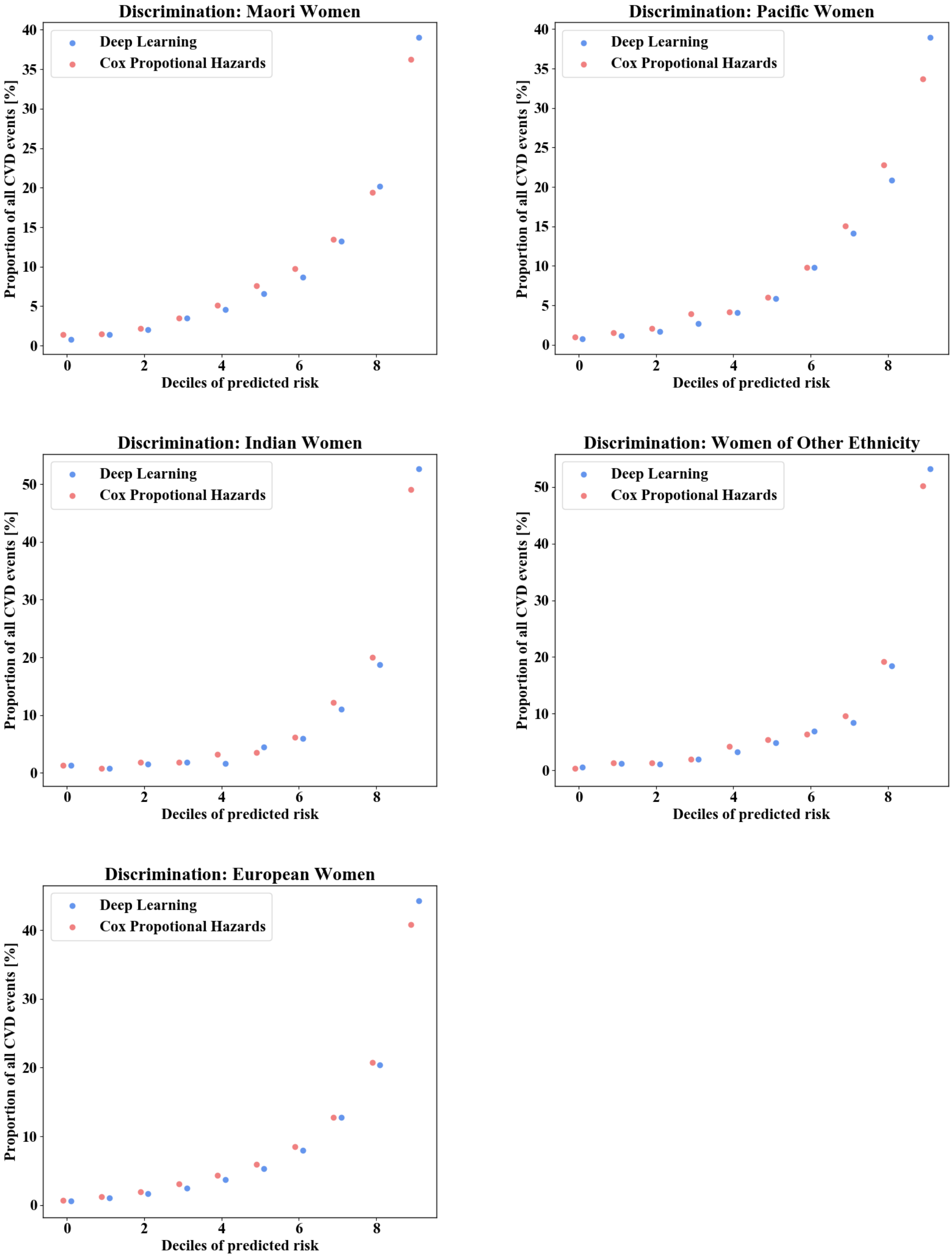

Supplementary Figure S4. Discrimination in women stratified by ethnicity.

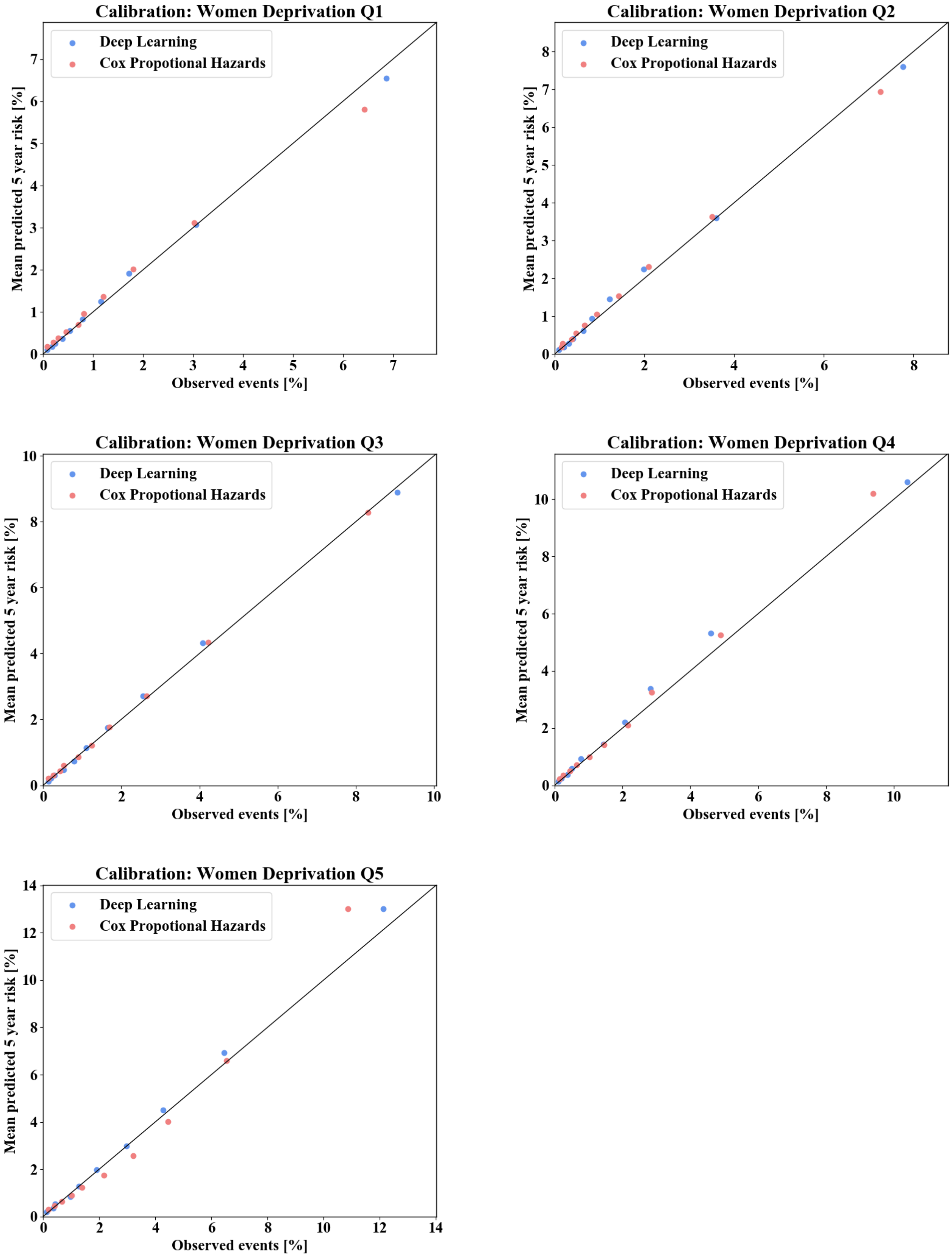

Supplementary Figure S5. Calibration in women stratified by quintile of deprivation.

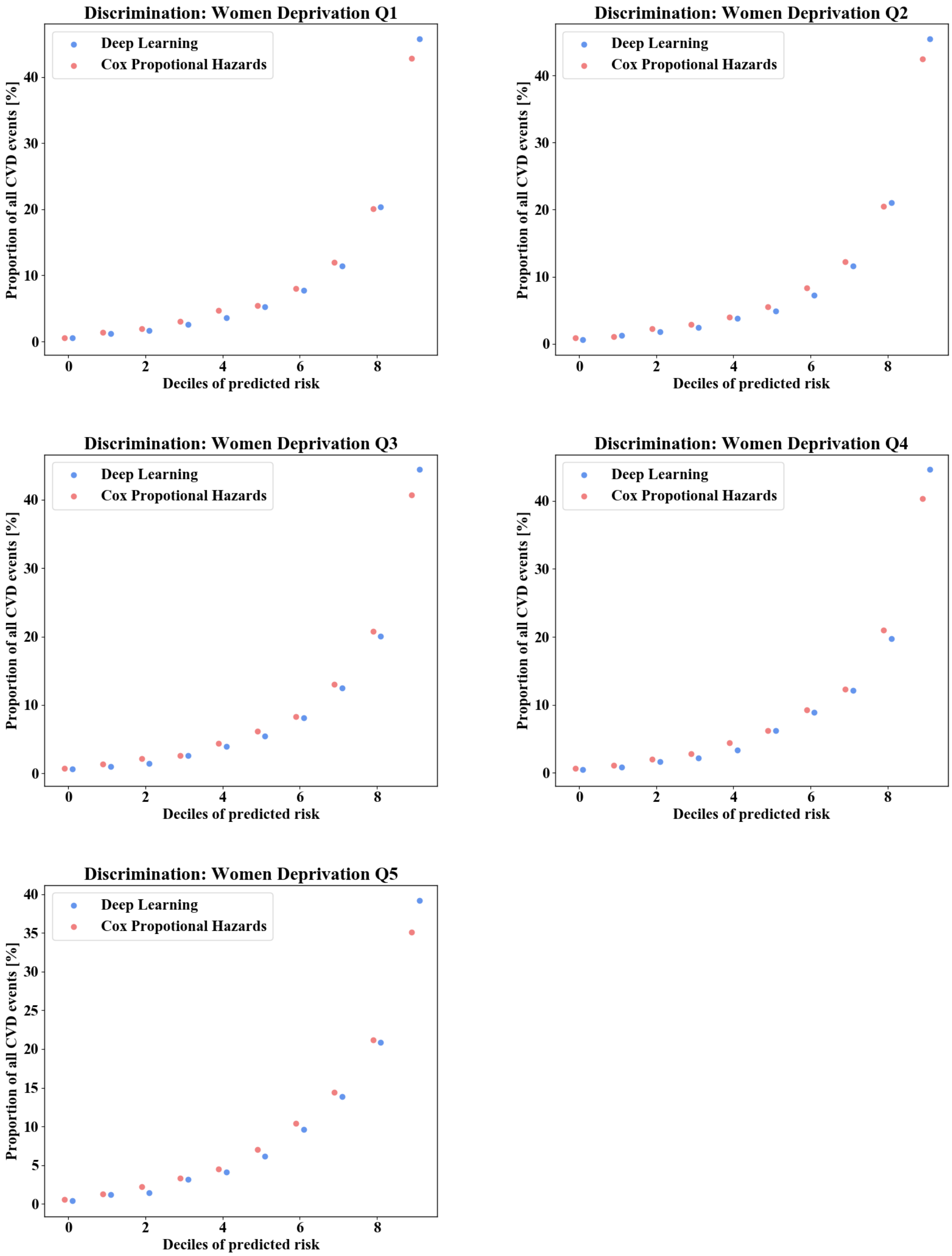

Supplementary Figure S6. Discrimination in women stratified by quintile of deprivation.

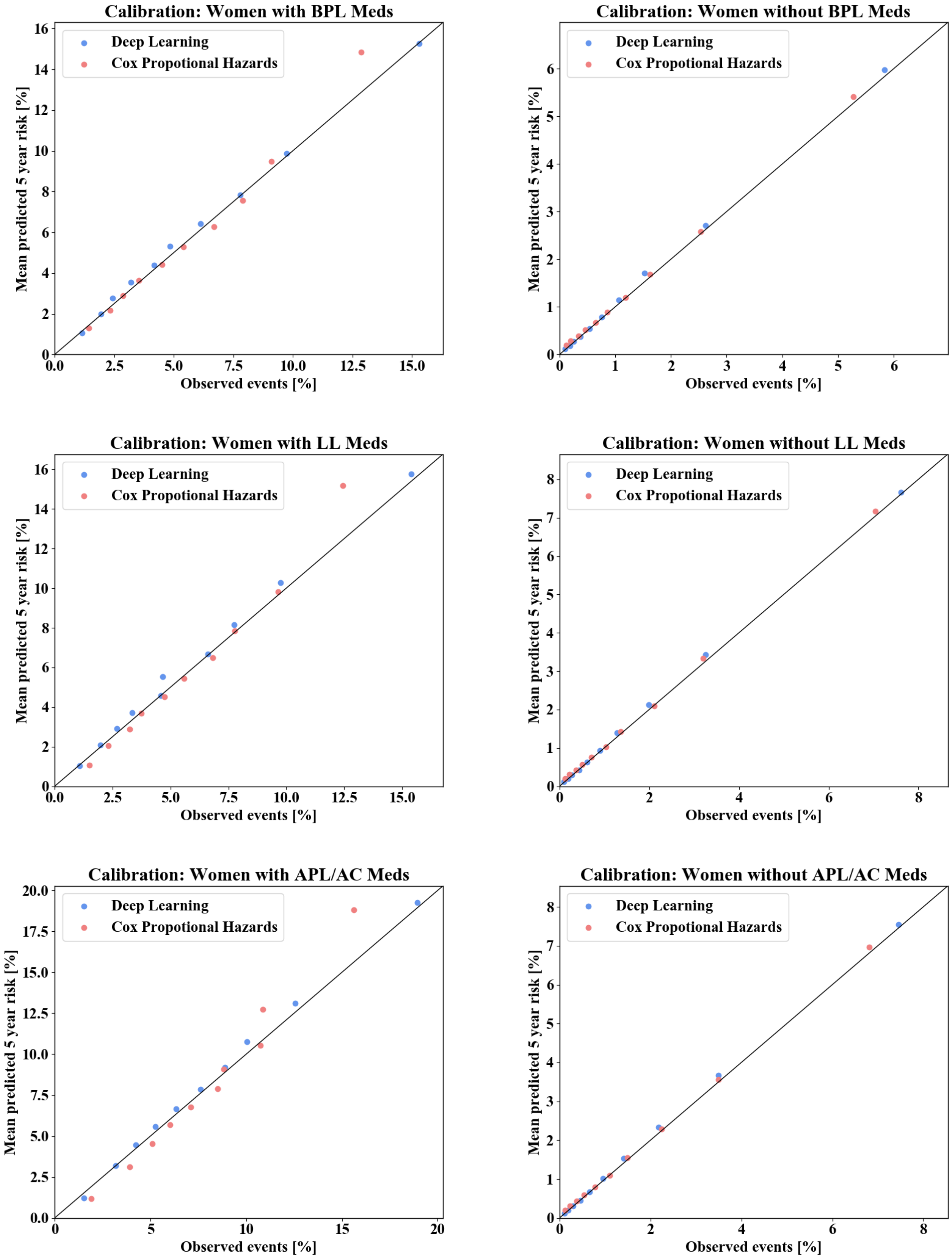

Supplementary Figure S7. Calibration in women stratified by dispensing of preventive medications. BPL Meds: blood-pressure-lowering medications; LL Meds: lipid-lowering medications; APL/AC Meds: antiplatelet/anticoagulant medications.

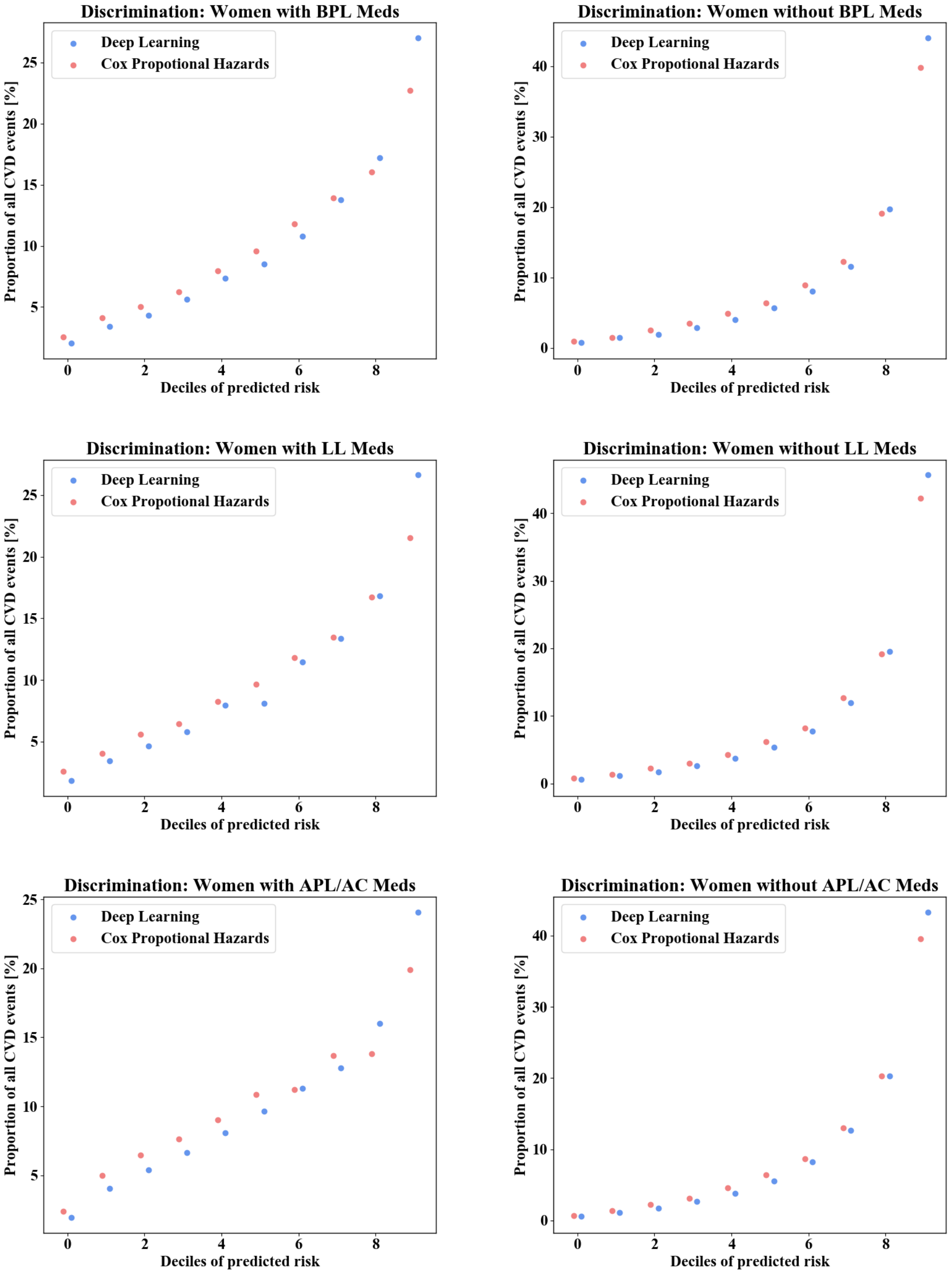

Supplementary Figure S8. Discrimination in women stratified by dispensing of preventive medications. BPL Meds: blood-pressure-lowering medications; LL Meds: lipid-lowering medications; APL/AC Meds: antiplatelet/anticoagulat medications.

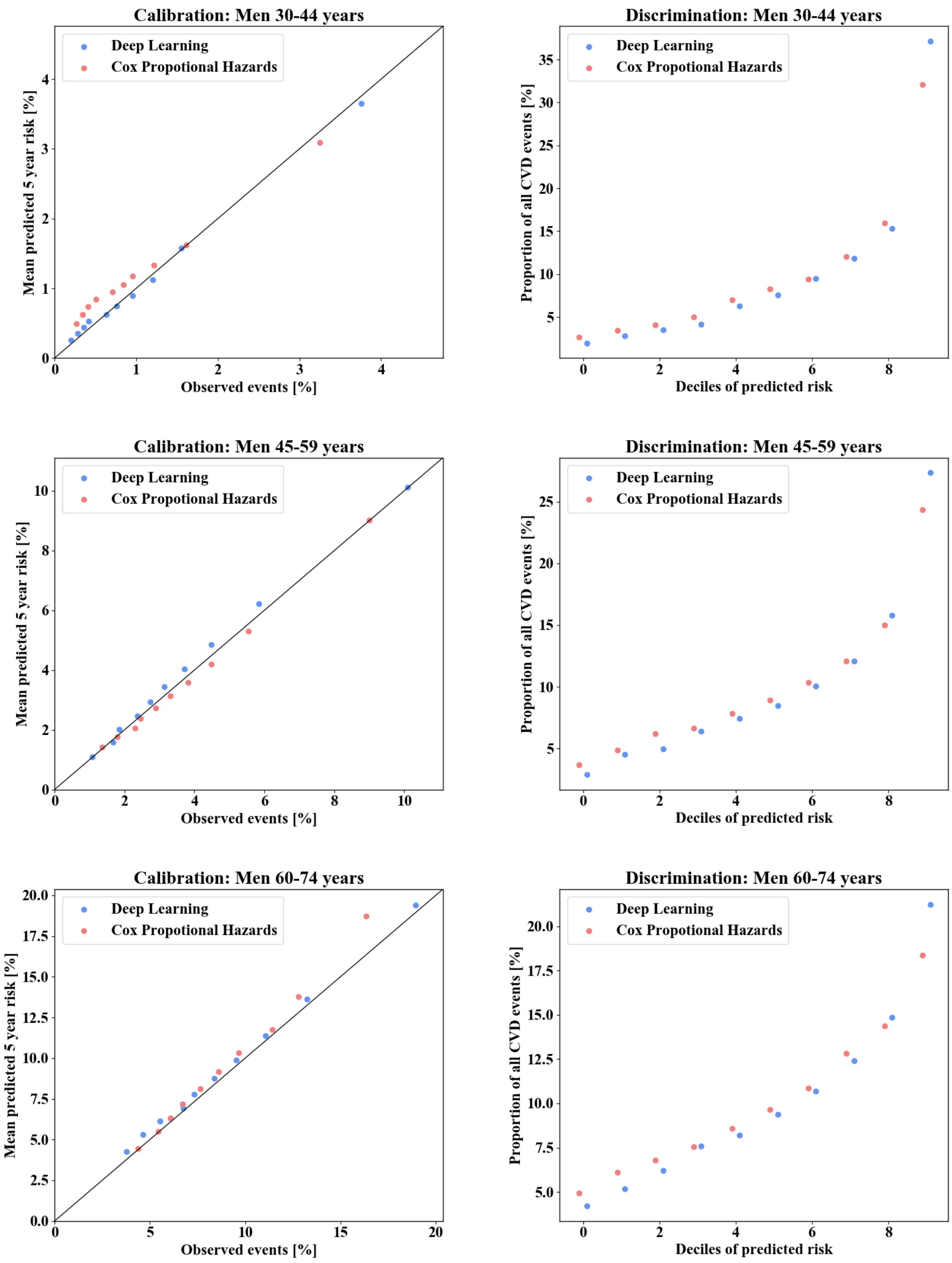

Supplementary Figure S9. Calibration and discrimination in men stratified by 15-year age bands.

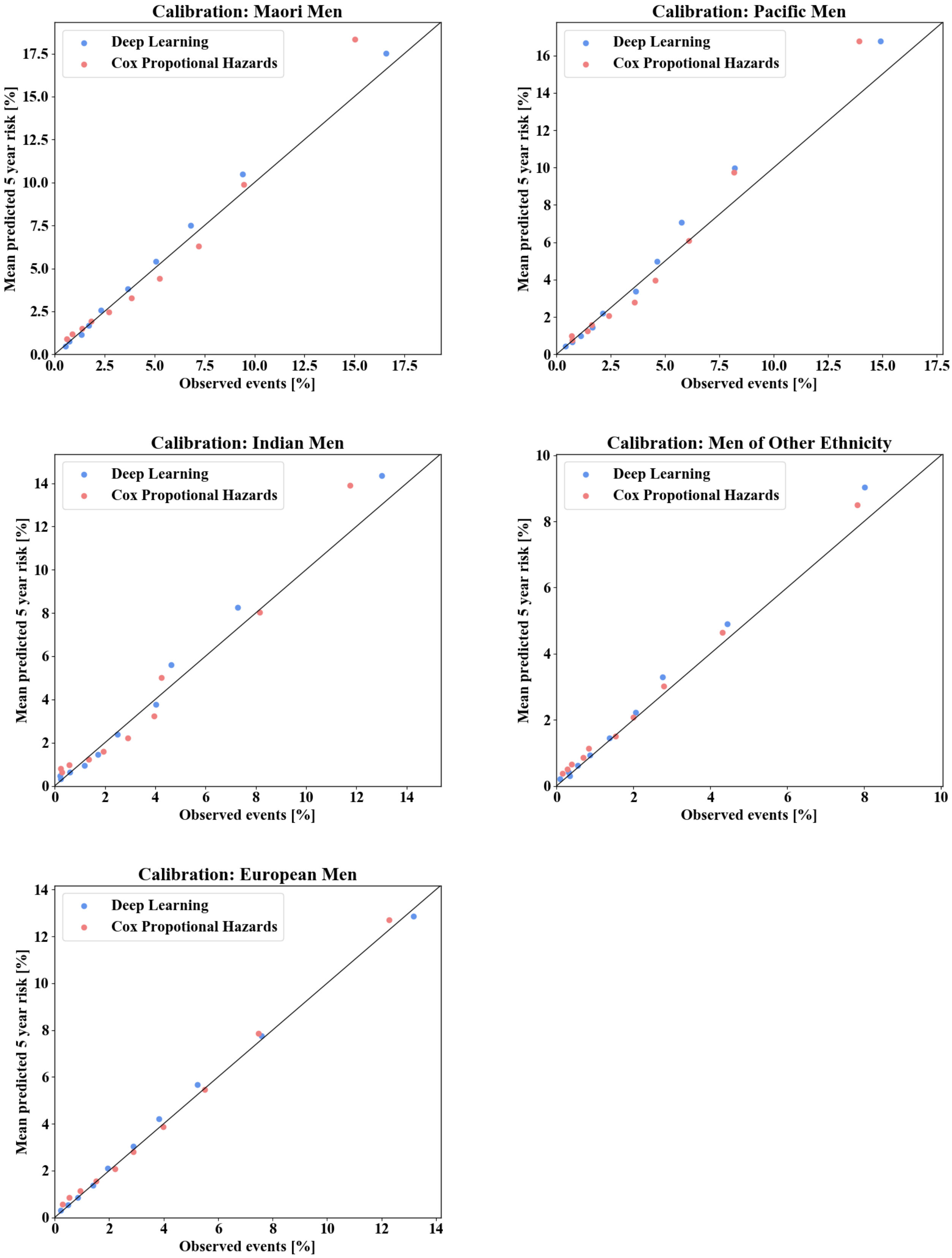

Supplementary Figure S10. Calibration in men stratified by ethnicity.

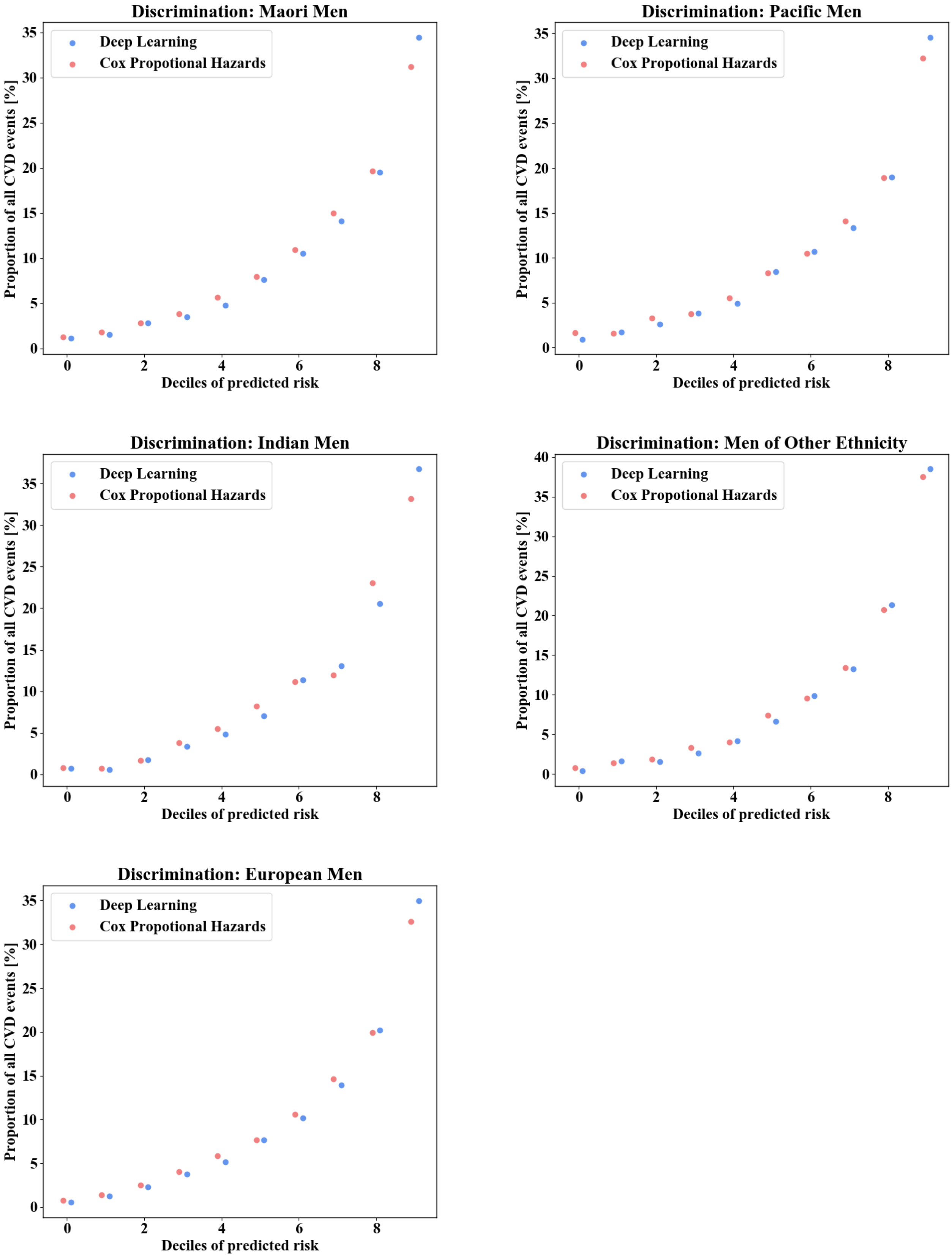

Supplementary Figure S11. Discrimination in men stratified by ethnicity.

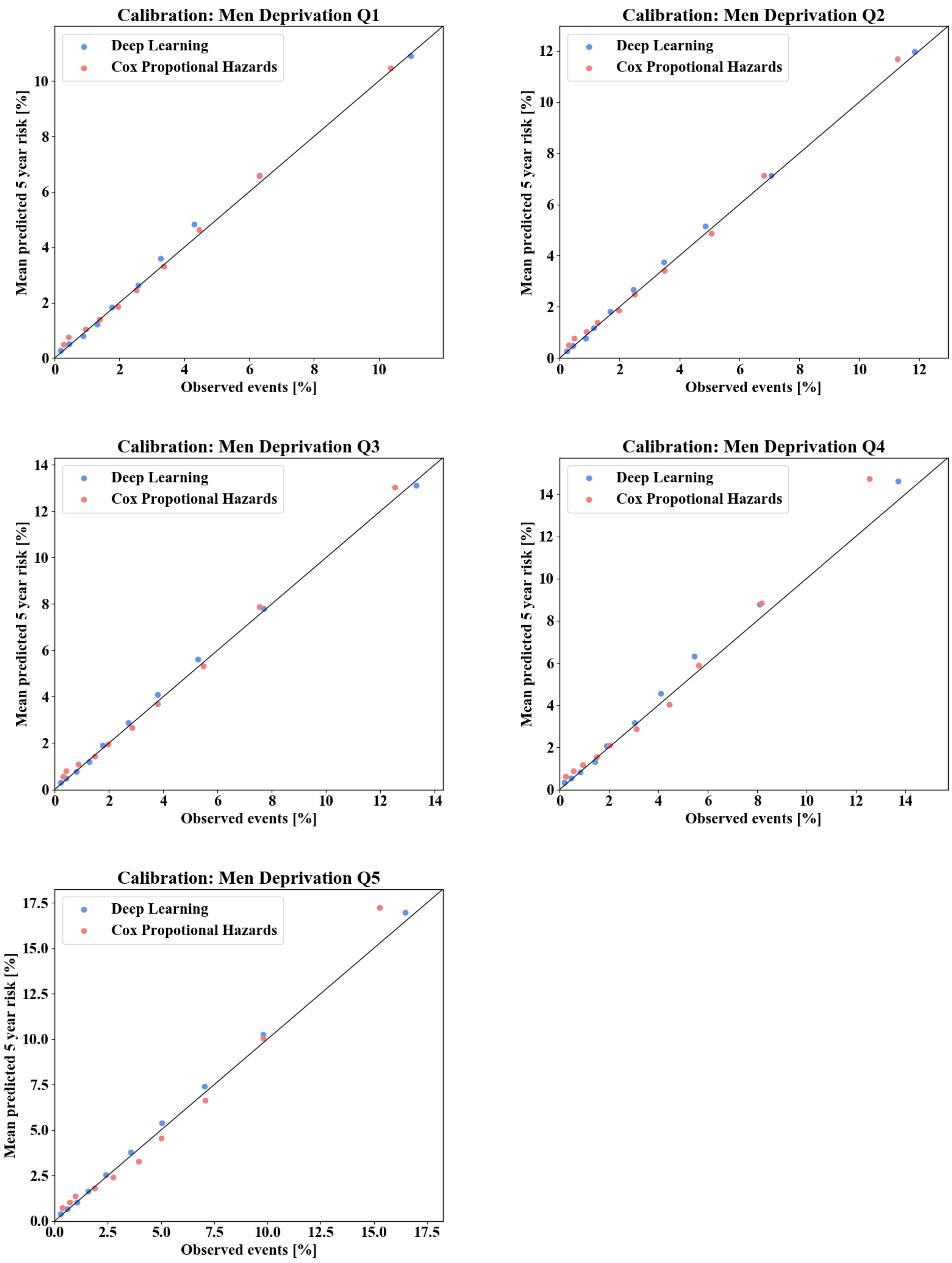

Supplementary Figure S12. Calibration in men stratified by quintile of deprivation.

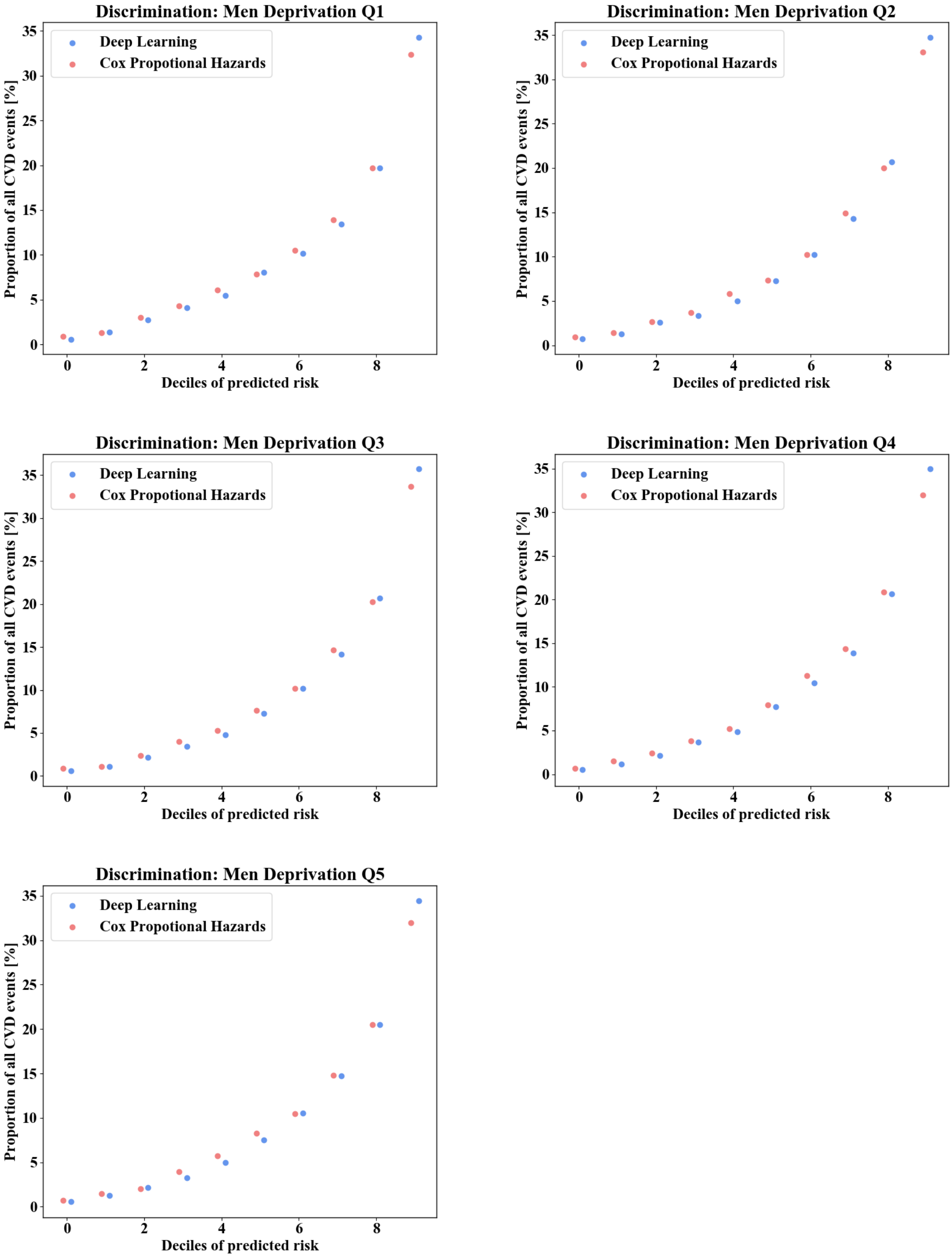

Supplementary Figure S13. Discrimination in men stratified by quintile of deprivation.

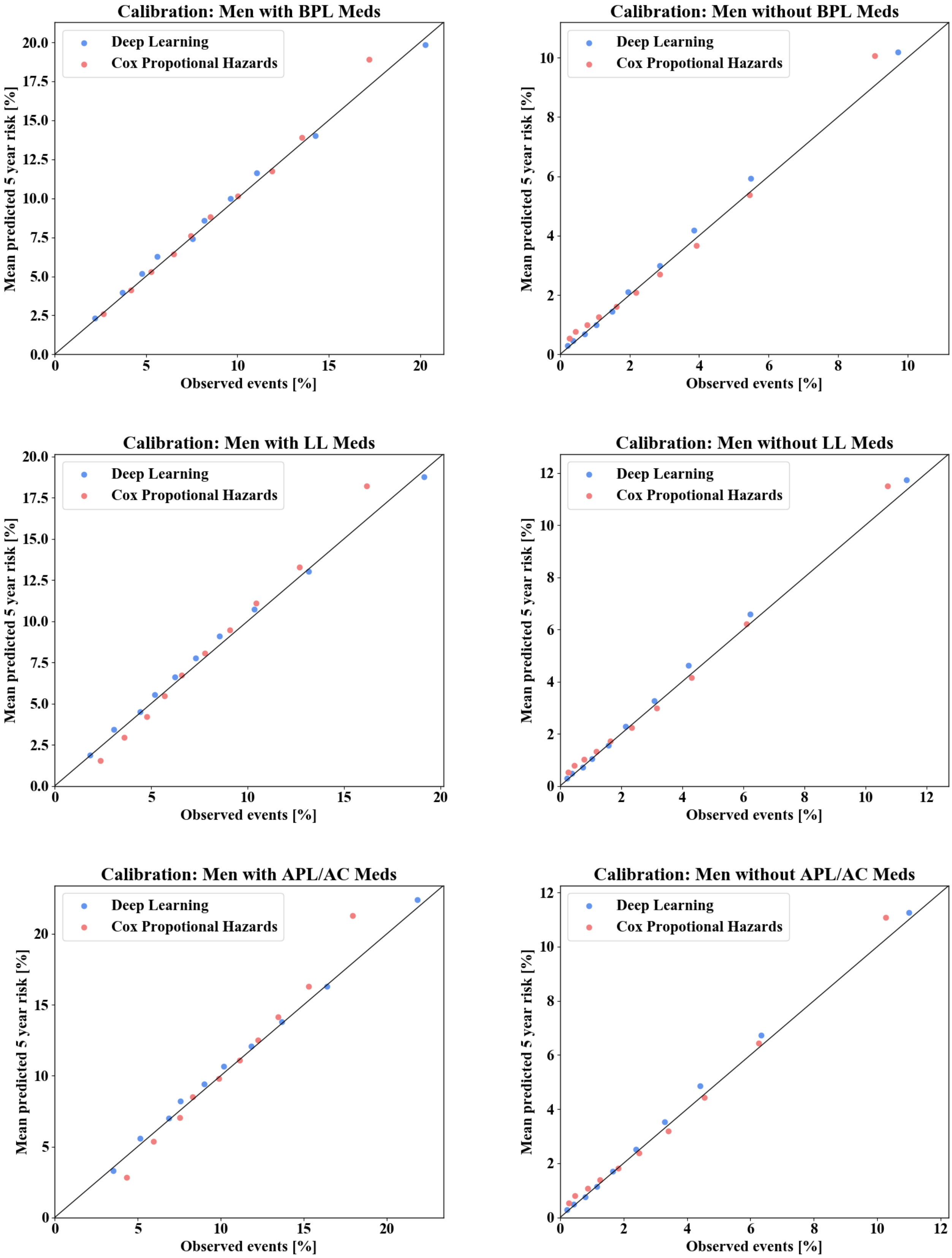

Supplementary Figure S14. Calibration in men stratified by dispensing of preventive medications. BPL Meds: blood-pressure-lowering medications; LL Meds: lipid-lowering medications; APL/AC Meds: antiplatelet/anticoagulat medications.

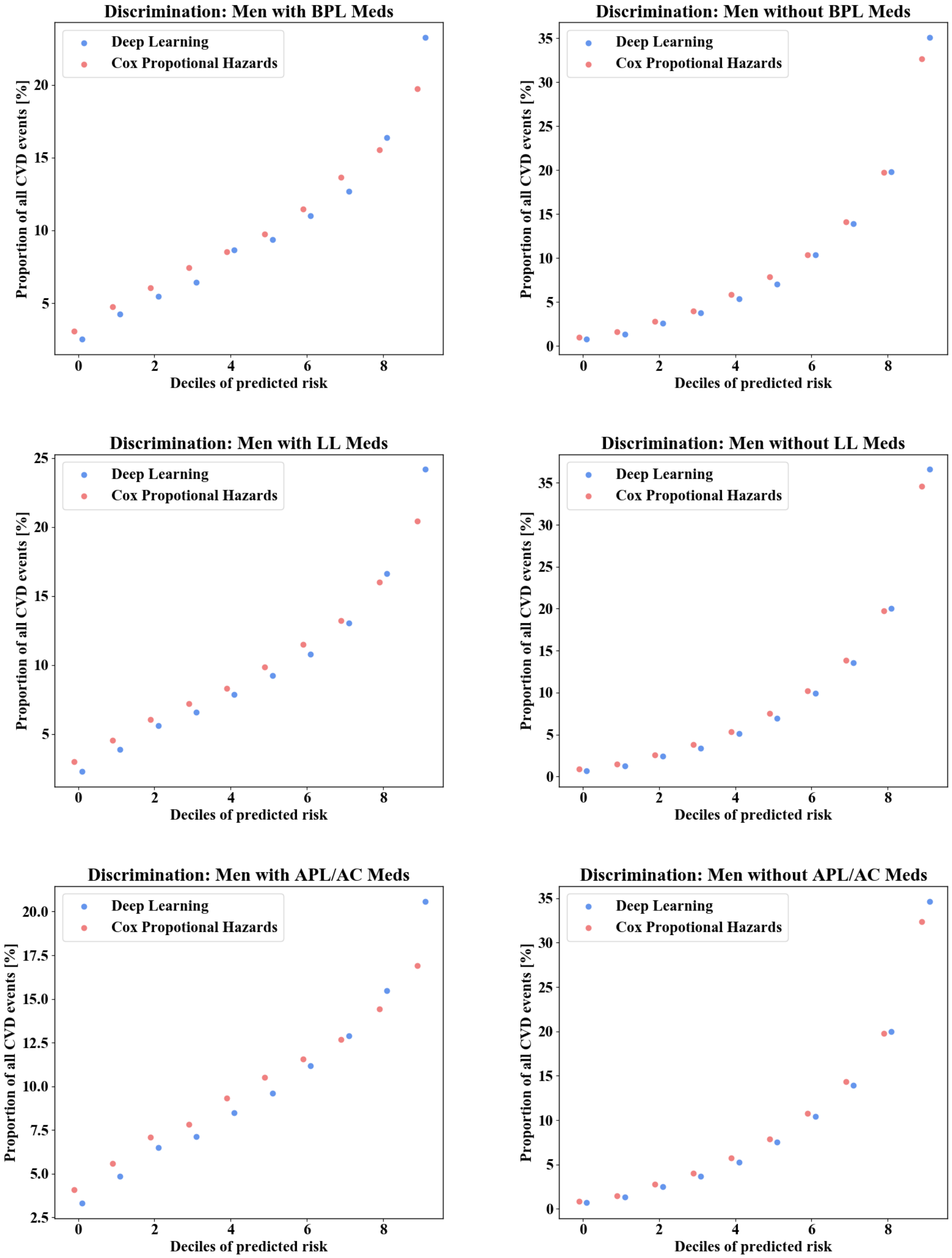

Supplementary Figure S15. Discrimination in men stratified by dispensing of preventive medications. BPL Meds: blood-pressure-lowering medications; LL Meds: lipid-lowering medications; APL/AC Meds: antiplatelet/anticoagulat medications.

SUPPLEMENTARY REFERENCES

1. Ministry of Health (New Zealand). ICD-10-AM/ACHI/ACS [Internet]. 2019 [cited 2020 Nov 1]. Available from: https://www.health.govt.nz/nz-health-statistics/classification-and-terminology/icd-10-am-achi-acs

2. Kvamme H, Borgan Ø, Scheel I. Time-to-event prediction with neural networks and Cox regression. Journal of machine learning research. 2019;20(129):1–30.

3. Cho K, Van Merriënboer B, Gulcehre C, Bahdanau D, Bougares F, Schwenk H, et al. Learning phrase representations using RNN encoder-decoder for statistical machine translation. ArXiv:14061078. 2014; preprint: not peer reviewed.

4. Hochreiter S, Schmidhuber J. Long short-term memory. Neural computation. 1997;9(8):1735–80.

5. Srivastava N, Hinton G, Krizhevsky A, Sutskever I, Salakhutdinov R. Dropout: a simple way to prevent neural networks from overfitting. The journal of machine learning research. 2014;15(1):1929–58.

6. Yang Z, Yang D, Dyer C, He X, Smola A, Hovy E. Hierarchical attention networks for document classification. In: Proceedings of the 2016 conference of the North American chapter of the association for computational linguistics: human language technologies. 2016. p. 1480–9.

7. Bergstra JS, Bardenet R, Bengio Y, Kégl B. Algorithms for hyper-parameter optimization. In: Advances in neural information processing systems. 2011. p. 2546–54.

8. Jamieson K, Talwalkar A. Non-stochastic best arm identification and hyperparameter optimization. In: Artificial Intelligence and Statistics. 2016. p. 240–8.

9. Royston P, Sauerbrei W. A new measure of prognostic separation in survival data. Statistics in medicine. 2004;23(5):723–48.

10. Harrell FE, Califf RM, Pryor DB, Lee KL, Rosati RA. Evaluating the yield of medical tests. Jama. 1982;247(18):2543–6.

11. Graf E, Schmoor C, Sauerbrei W, Schumacher M. Assessment and comparison of prognostic classification schemes for survival data. Statistics in medicine. 1999;18(17–18):2529–45.

12. Blattenberger G, Lad F. Separating the Brier score into calibration and refinement components: A graphical exposition. The American Statistician. 1985;39(1):26–32.

13. Alpaydm E. Combined 5x2 cv F test for comparing supervised classification learning algorithms. Neural computation. 1999;11(8):1885–92.
